# Personalized schedules for surveillance of low‐risk prostate cancer patients

**DOI:** 10.1111/biom.12940

**Published:** 2018-07-23

**Authors:** Anirudh Tomer, Daan Nieboer, Monique J. Roobol, Ewout W. Steyerberg, Dimitris Rizopoulos

**Affiliations:** ^1^ Department of Biostatistics Erasmus University Medical Center The Netherlands; ^2^ Department of Public Health Erasmus University Medical Center The Netherlands; ^3^ Department of Urology Erasmus University Medical Center The Netherlands; ^4^ Department of Medical Statistics and Bioinformatics Leiden University Medical Center The Netherlands

**Keywords:** Active surveillance, Biopsy, Joint models, Personalized medicine, Prostate cancer

## Abstract

Low‐risk prostate cancer patients enrolled in active surveillance (AS) programs commonly undergo biopsies on a frequent basis for examination of cancer progression. AS programs employ a fixed schedule of biopsies for all patients. Such fixed and frequent schedules may schedule unnecessary biopsies. Since biopsies are burdensome, patients do not always comply with the schedule, which increases the risk of delayed detection of cancer progression. Motivated by the world's largest AS program, Prostate Cancer Research International Active Surveillance (PRIAS), we present personalized schedules for biopsies to counter these problems. Using joint models for time‐to‐event and longitudinal data, our methods combine information from historical prostate‐specific antigen levels and repeat biopsy results of a patient, to schedule the next biopsy. We also present methods to compare personalized schedules with existing biopsy schedules.

## 1 Introduction

Prostate cancer (PCa) is the second most frequently diagnosed cancer (14% of all cancers) in males worldwide (Torre et al., 2015). The increase in diagnosis of low‐grade PCa has been attributed to increase in life expectancy and increase in the number of screening programs (Potosky et al., 1995). An issue of screening programs that has also been established in other types of cancers (e.g., breast cancer) is over‐diagnosis. To avoid overtreatment, patients diagnosed with low‐grade PCa are commonly advised to join active surveillance (AS) programs. In order to delay serious treatments such as surgery, chemotherapy, or radiotherapy, in AS PCa progression is routinely examined via serum prostate‐specific antigen (PSA) levels, digital rectal examination, medical imaging, and biopsy etc.

Biopsies are the most painful, prone to medical complications (Loeb et al., 2013) and yet also the most reliable PCa progression examination technique used in AS. When a patient's biopsy Gleason grading becomes larger than 6 (Gleason reclassification or GR), he is advised to switch from AS to active treatment (Bokhorst et al., 2015). Hence the timing of biopsies has significant medical implications. The world's largest AS program, Prostate Cancer Research International Active Surveillance (PRIAS) conducts biopsies at year 1, 4, 7, and 10 of follow‐up, and every 5 years thereafter. However, it switches to a more frequent, annual biopsy schedule for faster‐progressing patients. These are patients with PSA doubling time (PSA‐DT) between 0 and 10 years, which is measured as the inverse of the slope of the regression line through the base two logarithm of PSA values. In contrast, many AS programs use annual schedule for all patients (Tosoian et al., 2011; Welty et al., 2015). Consequently, for slowly‐progressing PCa patients many unnecessary biopsies are scheduled. Furthermore, patients may not always comply with such schedules (Bokhorst et al., 2015), which can lead to delayed detection of PCa and reduce the effectiveness of AS.

This article is motivated by the need to reduce the medical burden of repeat biopsies while simultaneously avoiding late detection of PCa progression. To this end, we intend to develop personalized schedules for biopsies using historical PSA measurements and biopsy results of patients. Personalized schedules for screening have received much interest in the literature, especially in the medical decision making context. For example, Markov decision process (MDP) models have been used to create personalized screening schedules for diabetic retinopathy (Bebu and Lachin, 2017), breast cancer (Ayer et al., 2012), cervical cancer (Akhavan‐Tabatabaei et al., 2017), and colorectal cancer (Erenay et al., 2014). Another type of model called joint model for time‐to‐event and longitudinal data (Tsiatis and Davidian, 2004; Rizopoulos, 2012) has also been used to create personalized schedules for the measurement of longitudinal biomarkers (Rizopoulos et al., 2016). In the context of PCa, Zhang et al. (2012) have used partially observable MDP models to personalize the decision of (not) deferring a biopsy to the next check‐up time during the screening process. This decision is based on the baseline characteristics as well as a discretized PSA level of the patient at the current check‐up time.

In comparison to the work referenced above, the schedules we propose in this article account for the latent between‐patient heterogeneity. We achieve this by using joint models, which are inherently patient‐specific because they utilize random effects. Secondly, joint models allow a continuous time scale and utilize the entire history of PSA levels. Lastly, instead of making a binary decision of (not) deferring a biopsy to the next pre‐scheduled check‐up time, we schedule biopsies at a per‐patient optimal future time. To this end, using joint models we first obtain a full specification of the joint distribution of PSA levels and time of GR. We then use it to define a patient‐specific posterior predictive distribution of the time of GR, given the observed PSA measurements and repeat biopsies up to the current check‐up time. Using the general framework of Bayesian decision theory, we propose a set of loss functions which are minimized to find the optimal time of conducting a biopsy. These loss functions yield us two categories of personalized schedules, those based on expected time of GR and those based on the risk of GR. In addition, we analyze an approach where the two types of schedules are combined. We also present methods to evaluate and compare the various schedules for biopsies.

The rest of the article is organized as follows. Section 2 briefly covers the joint modeling framework. Section 3 details the personalized scheduling approaches we have proposed in this article. In Section 4, we discuss methods for evaluation and selection of a schedule. In Section 5, we demonstrate the personalized schedules by employing them for the patients from the PRIAS program. Lastly, in Section 6, we present the results of a simulation study we conducted to compare personalized schedules with PRIAS and annual schedule.

## 2 Joint Model for Time‐to‐Event and Longitudinal Outcomes

We start with a short introduction of the joint modeling framework we will use in our following developments. Let $T_{i}^{*}$ denote the true GR time for the *i*‐th patient and let *S* be the schedule of his biopsies. Let the vector of the time of biopsies be denoted by $T_{i}^{S}=\{T_{i0}^{S},T_{i1}^{S},\ldots ,T_{iN_{i}^{S}}^{S};T_{\mathrm{ij}}^{S}<T_{\mathrm{ik}}^{S},\forall j<k\}$, where $N_{i}^{S}$ are the total number of biopsies conducted. Because biopsy schedules are periodical, $T_{i}^{*}$ cannot be observed directly and it is only known to fall in an interval $l_{i}<T_{i}^{*}\leq r_{i}$, where $l_{i}=T_{iN_{i}^{S}-1}^{S},r_{i}=T_{iN_{i}^{S}}^{S}$ if GR is observed, and $l_{i}=T_{iN_{i}^{S}}^{S},r_{i}=\infty$ if GR is not observed yet. Further let $y_{i}$ denote the $n_{i}\times 1$ vector of PSA levels for the *i*‐th patient. For a sample of *n* patients the observed data is denoted by $D_{n}=\{l_{i},r_{i},y_{i};i=1,\ldots ,n\}$.

The longitudinal outcome of interest, namely PSA level, is continuous in nature and thus to model it the joint model utilizes a linear mixed effects model (LMM) of the form:

$$\begin{array}{cc} y_{i}(t) & =m_{i}(t)+\epsilon_{i}(t)\\ & =x_{i}^{T}(t)\beta +z_{i}^{T}(t)b_{i}+\epsilon_{i}(t), \end{array}$$

where $x_{i}(t)$ and $z_{i}(t)$ denote the row vectors of the design matrix for fixed and random effects, respectively. The fixed and random effects are denoted by β and $b_{i}$, respectively. The random effects are assumed to be normally distributed with mean zero and q×q covariance matrix D. The true and unobserved, error free PSA level at time *t* is denoted by $m_{i}(t)$. The error $\epsilon_{i}(t)$ is assumed to be *t*‐distributed with three degrees of freedom and scale σ (see Web Appendix C.1), and is independent of the random effects $b_{i}$.

To model the effect of PSA on hazard of GR, joint models utilize a relative risk sub‐model. The hazard of GR for patient *i* at any time point *t*, denoted by $h_{i}(t)$, depends on a function of subject specific linear predictor $m_{i}(t)$ and/or the random effects:

$$\begin{array}{ccc} h_{i}(t\;|\;M_{i}(t),\;w_{i})\; & = & \;\underset{\Delta t\to 0}{\text{lim}}\frac{\mathrm{Pr}\{t\leq T_{i}^{*}<t+\Delta t|T_{i}^{*}\geq t,M_{i}(t),w_{i}\}}{\Delta t}\\ & = & h_{0}(t)\text{exp}[\gamma^{T}w_{i}+f\{M_{i}(t),b_{i},\alpha \}],\;t>0, \end{array}$$

where $M_{i}(t)=\{m_{i}(v),0\leq v\leq t\}$ denotes the history of the underlying PSA levels up to time *t*. The vector of baseline covariates is denoted by $w_{i}$, and γ are the corresponding parameters. The function f(·) parametrized by vector α specifies the functional form of PSA levels (Brown, 2009; Rizopoulos, 2012; Taylor et al., 2013; Rizopoulos et al., 2014) that is used in the linear predictor of the relative risk model. Some functional forms relevant to the problem at hand are the following:

$$\left\{\begin{array}{c} f\{M_{i}(t),b_{i},\alpha \}=\alpha m_{i}(t),\\ f\{M_{i}(t),b_{i},\alpha \}=\alpha_{1}m_{i}(t)+\alpha_{2}m_{i}^{\prime }(t),\;\mathrm{with}\;m_{i}^{\prime }(t)=\frac{dm_{i}(t)}{dt}. \end{array}\right.$$

These formulations of f(·) postulate that the hazard of GR at time *t* may be associated with the underlying level $m_{i}(t)$ of the PSA at *t*, or with both the level and velocity $m_{i}^{\prime }(t)$ of the PSA at *t*. Lastly, $h_{0}(t)$ is the baseline hazard at time *t*, and is modeled flexibly using P‐splines. The detailed specification of the baseline hazard, and parameter estimation using the Bayesian approach are presented in Web Appendix A of the supplementary material.

## 3 Personalized Schedules for Repeat Biopsies

We intend to use the joint model fitted to $D_{n}$, to create personalized schedules of biopsies. To this end, let us assume that a schedule is to be created for a new patient *j*, who is not present in $D_{n}$. Let *t* be the time of his latest biopsy, and $Y_{j}(s)$ denote his historical PSA measurements up to time *s*. The goal is to find the optimal time u>max(t,s) of the next biopsy.

### 3.1 Posterior Predictive Distribution for Time to GR

The information from $Y_{j}(s)$ and repeat biopsies is manifested by the posterior predictive distribution $g(T_{j}^{*})$, given by (baseline covariates $w_{i}$ are not shown for brevity hereafter):

$$\begin{array}{ccc} g(T_{j}^{*}) & = & p\{T_{j}^{*}|T_{j}^{*}>t,Y_{j}(s),D_{n}\}\\ & = & \int p\{T_{j}^{*}|T_{j}^{*}>t,Y_{j}(s),\theta \}p(\theta |D_{n})d\theta \\ & = & \int \int p(T_{j}^{*}|T_{j}^{*}>t,b_{j},\theta )\\ & & p\{b_{j}|T_{j}^{*}>t,Y_{j}(s),\theta \}p(\theta |D_{n})db_{j}d\theta . \end{array}$$

The distribution $g(T_{j}^{*})$ depends on $Y_{j}(s)$ and $D_{n}$ via the posterior distribution of random effects $b_{j}$ and posterior distribution of the vector of all parameters θ, respectively.

### 3.2 Loss Functions

To find the time *u* of the next biopsy, we use principles from statistical decision theory in a Bayesian setting (Berger, 1985; Robert, 2007). More specifically, we propose to choose *u* by minimizing the posterior expected loss $E_{g}\{L(T_{j}^{*},u)\}$, where the expectation is taken with respect to $g(T_{j}^{*})$. The former is given by:

$$E_{g}\{L(T_{j}^{*},u)\}=\int_{t}^{\infty }L(T_{j}^{*},u)p\{T_{j}^{*}|T_{j}^{*}>t,Y_{j}(s),D_{n}\}dT_{j}^{*}.$$

Various loss functions $L(T_{j}^{*},u)$ have been proposed in literature (Robert, 2007). The ones we utilize, and the corresponding motivations are presented next.

Given the burden of biopsies, ideally only one biopsy performed at the exact time of GR is sufficient. Hence, neither a time which overshoots the true GR time $T_{j}^{*}$, nor a time which undershoots it, is preferred. In this regard, the squared loss function $L(T_{j}^{*},u)={\left(T_{j}^{*}-u\right)}^{2}$ and the absolute loss function $L(T_{j}^{*},u)=\left|T_{j}^{*}-u\right|$ have the properties that the posterior expected loss is symmetric on both sides of $T_{j}^{*}$. Secondly, both loss functions have well known solutions available. The posterior expected loss for the squared loss function is given by:

(1) $$\begin{array}{ccc} E_{g}\{L(T_{j}^{*},u)\} & = & E_{g}\{{\left(T_{j}^{*}-u\right)}^{2}\}\\ & = & E_{g}\{{\left(T_{j}^{*}\right)}^{2}\}+u^{2}-2{\mathrm{uE}}_{g}(T_{j}^{*}). \end{array}$$

The posterior expected loss in (1) attains its minimum at $u=E_{g}(T_{j}^{*})$, that is, the expected time of GR. The posterior expected loss for the absolute loss function is given by:

(2) $$\begin{array}{ccc} E_{g}\{L(T_{j}^{*},u)\} & = & E_{g}(\left|T_{j}^{*}-u\right|)\\ & = & \;\;\;\int_{u}^{\infty }\;\;(T_{j}^{*}-u)g(T_{j}^{*})dT_{j}^{*}+\int_{t}^{u}\;\;(u-T_{j}^{*})g(T_{j}^{*})dT_{j}^{*}. \end{array}$$

The posterior expected loss in (2) attains its minimum at $u={\mathrm{median}}_{g}(T_{j}^{*})$, that is, the median time of GR. It can also be expressed as $\pi_{j}^{-1}(0.5|t,s)$, where $\pi_{j}^{-1}(\cdot )$ is the inverse of dynamic survival probability $\pi_{j}(u|t,s)$ of patient *j* (Rizopoulos, 2011). It is given by:

$$\pi_{j}(u|t,s)=\mathrm{Pr}\{T_{j}^{*}\geq u|T_{j}^{*}>t,Y_{j}(s),D_{n}\},\;u\geq t.$$

Even though $E_{g}(T_{j}^{*})$ or ${\mathrm{median}}_{g}(T_{j}^{*})$ may be obvious choices from a statistical perspective, from the viewpoint of doctors or patients, it could be more intuitive to make the decision for the next biopsy by placing a cutoff 1−κ, where 0≤κ≤1, on the dynamic incidence/risk of GR. This approach would be successful if κ can sufficiently well differentiate between patients who will obtain GR in a given period of time versus others. This approach is also useful when patients are apprehensive about delaying biopsies beyond a certain risk cutoff. Thus, a biopsy can be scheduled at a time point *u* such that the dynamic risk of GR is higher than a certain threshold 1−κ,  beyond *u*. To this end, the posterior expected loss for the following multilinear loss function can be minimized to find the optimal *u*:

$$\begin{array}{ccc} L_{k_{1},k_{2}}(T_{j}^{*},u)=\left\{\begin{array}{cc} k_{2}(T_{j}^{*}-u),k_{2}>0 & \;\mathrm{if}\;T_{j}^{*}>u,\\ k_{1}(u-T_{j}^{*}),k_{1}>0 & \;\mathrm{otherwise}, \end{array}\right. & & \end{array}$$

where $k_{1},k_{2}$ are constants parameterizing the loss function. The posterior expected loss $E_{g}\{L_{k_{1},k_{2}}(T_{j}^{*},u)\}$ obtains its minimum at $u=\pi_{j}^{-1}\{k_{1}/\left(k_{1}+k_{2}\right)|t,s\}$ (Robert, 2007). The choice of the two constants $k_{1}$ and $k_{2}$ is equivalent to the choice of $\kappa =k_{1}/\left(k_{1}+k_{2}\right)$.

In practice, for some patients, we may not have sufficient information to accurately estimate their PSA profile. The resulting high variance of $g(T_{j}^{*})$ could lead to a mean (or median) time of GR which overshoots the true $T_{j}^{*}$ by a big margin. In such cases, the approach based on the dynamic risk of GR with smaller risk thresholds is more risk‐averse and thus could be more robust to large overshooting margins. This consideration leads us to a hybrid approach, namely, to select *u* using dynamic risk of GR‐based approach when the spread of $g(T_{j}^{*})$ is large, while using $E_{g}(T_{j}^{*})$ or ${\mathrm{median}}_{g}(T_{j}^{*})$ when the spread of $g(T_{j}^{*})$ is small. What constitutes a large spread will be application‐specific. In PRIAS, within the first 10 years, the maximum possible delay in detection of GR is 3 years. Thus, we propose that if the difference between the 0.025 quantile of $g(T_{j}^{*})$, and $E_{g}(T_{j}^{*})$ or ${\mathrm{median}}_{g}(T_{j}^{*})$ is more than 3 years then proposals based on the dynamic risk of GR be used instead.

### 3.3 Estimation

Since there is no closed form solution available for $E_{g}(T_{j}^{*})$, for its estimation we utilize the following relationship between $E_{g}(T_{j}^{*})$ and $\pi_{j}(u|t,s)$:

(3) $$E_{g}(T_{j}^{*})=t+\int_{t}^{\infty }\pi_{j}(u|t,s)du.$$

However, as mentioned earlier, selection of the optimal biopsy time based on $E_{g}(T_{j}^{*})$ alone will not be practically useful when the ${\mathrm{var}}_{g}(T_{j}^{*})$ is large, which is given by:

(4) $${\mathrm{var}}_{g}(T_{j}^{*})=2\int_{t}^{\infty }(u-t)\pi_{j}(u|t,s)du-{\{\int_{t}^{\infty }\pi_{j}(u|t,s)du\}}^{2}.$$

Since there is no closed form solution available for the integrals in (3) and (4), we approximate them using Gauss‐Kronrod quadrature (see Web Appendix B). The variance depends both on the last biopsy time *t* and the PSA history $Y_{j}(s)$, as demonstrated in Section 5.2.

For schedules based on dynamic risk of GR, the choice of threshold κ has important consequences because it dictates the timing of biopsies. Often it may depend on the amount of risk that is acceptable to the patient (if maximum acceptable risk is 5%, κ=0.95). When κ cannot be chosen on the basis of the input of the patients, we propose to automate its choice. More specifically, given the time *t* of latest biopsy we propose to choose a κ for which a binary classification accuracy measure (López‐Ratón et al., 2014), discriminating between cases (patients who experience GR) and controls, is maximized. In joint models, a patient *j* is predicted to be a case in the time window Δt if $\pi_{j}(t+\Delta t|t,s)\leq \kappa$, or a control if $\pi_{j}(t+\Delta t|t,s)>\kappa$ (Rizopoulos, 2016; Rizopoulos et al., 2017). We choose Δt to be 1 year. This is because, in AS programs at any point in time, it is of interest to identify and provide extra attention to patients who may obtain GR in the next 1 year. As for the choice of the binary classification accuracy measure, we chose $F_{1}$ score since it is in line with our goal to focus on potential cases in time window Δt. The $F_{1}$ score combines both sensitivity and positive predictive value (PPV) and is defined as:

$$\begin{array}{ccc} F_{1}(t,\Delta t,s,\kappa ) & = & 2\frac{\mathrm{TPR}(t,\Delta t,s,\kappa )\;\mathrm{PPV}(t,\Delta t,s,\kappa )}{\mathrm{TPR}(t,\Delta t,s,\kappa )+\mathrm{PPV}(t,\Delta t,s,\kappa )},\\ \mathrm{TPR}(t,\Delta t,s,\kappa ) & = & \mathrm{Pr}\{\pi_{j}(t+\Delta t|t,s)\leq \kappa |t<T_{j}^{*}\leq t+\Delta t\},\\ \mathrm{PPV}(t,\Delta t,s,\kappa ) & = & \mathrm{Pr}\{t<T_{j}^{*}\leq t+\Delta t|\pi_{j}(t+\Delta t|t,s)\leq \kappa \}, \end{array}$$

where TPR(·) and PPV(·) denote time dependent true positive rate (sensitivity) and positive predictive value (precision), respectively. The estimation for both is similar to the estimation of AUC(t,Δt,s) given by Rizopoulos et al. (2017). Since a high $F_{1}$ score is desired, the corresponding value of κ is $\arg\;\max_{\kappa }F_{1}(t,\Delta t,s,\kappa )$. We compute the latter using a grid search approach. That is, first the $F_{1}$ score is computed using the available dataset over a fine grid of κ values between 0 and 1, and then κ corresponding to the highest $F_{1}$ score is chosen. Furthermore, in this article we use κ chosen only on the basis of the $F_{1}$ score.

### 3.4 Algorithm

**Figure 1:**
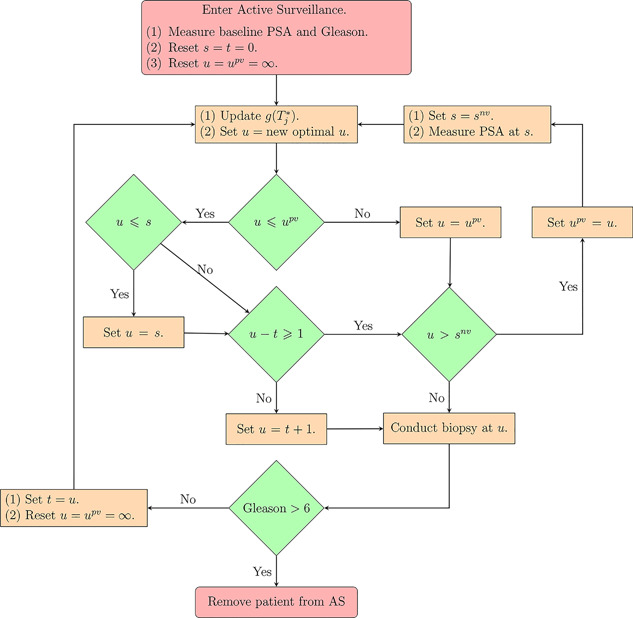
Algorithm for creating a personalized schedule for patient *j*. The time of the latest biopsy is denoted by *t*. The time of the latest available PSA measurement is denoted by *s*. The proposed personalized time of biopsy is denoted by *u*. The time at which a repeat biopsy was proposed on the last visit to the hospital is denoted by $u^{\mathrm{pv}}$. The time of the next visit for the measurement of PSA is denoted by $s^{\mathrm{nv}}$. This figure appears in color in the electronic version of this article.

When a biopsy gets scheduled at a time $u<T_{j}^{*}$, then GR is not detected at *u* and at least one more biopsy is required at an optimal time $u^{\mathrm{new}}>\max(u,s)$. This process is repeated until GR is detected. To aid in medical decision making, we elucidate this process via an algorithm in Figure 1. AS programs strongly advise that two biopsies have a gap of at least 1 year. Thus, when u−t<1, the algorithm postpones *u* to t+1, because it is the time nearest to *u*, at which the 1‐year gap condition is satisfied.

## 4 Evaluation of Schedules

In order to compare various schedules of biopsies, we require measures of their efficacy. We propose to use two measures, namely the number of biopsies (burden) $N_{j}^{S}\geq 1$ a schedule *S* conducts for the *j*‐th patient to detect GR, and the offset $O_{j}^{S}\geq 0$ by which it overshoots $T_{j}^{*}$. The offset $O_{j}^{S}$ is defined as $O_{j}^{S}=T_{jN_{j}^{S}}^{S}-T_{j}^{*}$, where $T_{jN_{j}^{S}}^{S}\geq T_{j}^{*}$ is the time at which GR is detected. Our interest lies in the joint distribution $p(N_{j}^{S},O_{j}^{S})$ of the number of biopsies and the offset. The least burdensome scenario is when $N_{j}^{S}=1$ and $O^{S}=0$. Hence, realistically we should select a schedule with a low mean number of biopsies $E(N_{j}^{S})$ as well a low mean offset $E(O_{j}^{S})$. It is also desired that a schedule has a low variance for both the number of biopsies $\mathrm{var}(N_{j}^{S})$, and offset $\mathrm{var}(O_{j}^{S})$, so that the schedule works similarly for most patients.

### 4.1 Choosing a Schedule

Given the multiple schedules of biopsies, it is of clinical interest to choose a suitable schedule. Using principles from compound optimal designs (Läuter, 1976), we propose to choose a schedule *S* which minimizes a loss function of the following form:

(5) $$L(S)=\sum_{r=1}^{R}\eta_{r}R_{r}(N_{j}^{S}),$$

where $R_{r}(\cdot )$ is a function of either $N_{j}^{S}$ or $O_{j}^{S}$ (for brevity, only $N_{j}^{S}$ is used in the equation above). Some examples of $R_{r}(\cdot )$ are mean, median, variance, and quantile function. Constants $\eta_{1},\ldots ,\eta_{R}$, where $0\leq \eta_{r}\leq 1$ and $\sum_{r=1}^{R}\eta_{r}=1$, are weights to differentially weigh‐in the contribution of each of the *R* criteria. An example loss function is:

(6) $$L(S)=\eta_{1}E(N_{j}^{S})+\eta_{2}E(O_{j}^{S}).$$

The choice of $\eta_{1}$ and $\eta_{2}$ is not easy, because the burden of a biopsy cannot be compared to a unit increase in offset easily. To obviate this problem we utilize the equivalence between compound and constrained optimal designs (Cook and Wong, 1994). More specifically, it can be shown that for any $\eta_{1}$ and $\eta_{2}$ there exists a constant C>0 for which minimization of the loss function in (6) is equivalent to minimization of the loss function subject to the constraint that $E(N_{j}^{S})<C$. That is, a schedule which conducts at most *C* biopsies on average and detects GR earliest should be chosen. The choice of *C* could be based on the number of biopsies a patient is willing to undergo. In the more generic case in (5), a schedule can be chosen by minimizing $R_{R}(\cdot )$ under the constraint $R_{r}(\cdot )<C_{r};r=1,\ldots ,R-1$.

## 5 Demonstration of Personalized Schedules

To demonstrate the personalized schedules, we apply them to the patients enrolled in PRIAS study. To this end, we divide the PRIAS dataset into a training part (5264 patients) and a demonstration part (three patients). We fit a joint model to the training dataset and then use it to create schedules for the demonstration patients. We fit the joint model using the R package **JMbayes** (Rizopoulos, 2016), which uses the Bayesian approach for parameter estimation.

### 5.1 Fitting the Joint Model to the PRIAS Dataset

For each of the PRIAS patients, we know their age at the time of inclusion in AS, PSA history, and the time interval in which GR is detected. For the longitudinal analysis of PSA we use ${\text{log}}_{2}(\mathrm{PSA}+1)$ measurements instead of the raw data (Pearson et al., 1994; Lin et al., 2000). The longitudinal sub‐model of the joint model we fit is given by:

(7) $$\begin{array}{ccc} {\text{log}}_{2}({\mathrm{PSA}}_{i}+1)(t) & = & \beta_{0}+\beta_{1}({\mathrm{Age}}_{i}-70)+\beta_{2}{\left({\mathrm{Age}}_{i}-70\right)}^{2}\\ & & +\sum_{k=1}^{4}\beta_{k+2}B_{k}(t,K)+b_{i0}+b_{i1}B_{7}(t,0.1)\\ & & +b_{i2}B_{8}(t,0.1)+\epsilon_{i}(t), \end{array}$$

where $B_{k}(t,K)$ denotes the *k*‐th basis function of a B‐spline with three internal knots at K={0.1,0.5,4} years, and boundary knots at 0 and 7 (0.99 quantile of the observed follow‐up times) years. The spline for the random effects consists of one internal knot at 0.1 years and boundary knots at 0 and 7 years. For the relative risk sub‐model the hazard function we fit is given by:

(8) $$\begin{array}{ccc} h_{i}(t) & = & h_{0}(t)\text{exp}\{\gamma_{1}({\mathrm{Age}}_{i}-70)+\gamma_{2}{\left({\mathrm{Age}}_{i}-70\right)}^{2}\\ & & +\alpha_{1}m_{i}(t)+\alpha_{2}m_{i}^{\prime }(t)\}, \end{array}$$

where $\alpha_{1}$ and $\alpha_{2}$ are measures of strength of the association between hazard of GR and ${\text{log}}_{2}({\mathrm{PSA}}_{i}+1)$ value $m_{i}(t)$ and ${\text{log}}_{2}({\mathrm{PSA}}_{i}+1)$ velocity $m_{i}^{\prime }(t)$, respectively.

From the fitted joint model we found that ${\text{log}}_{2}(\mathrm{PSA}+1)$ velocity and the age at the time of inclusion in AS were significantly associated with the hazard of GR. For any patient, an increase in ${\text{log}}_{2}(\mathrm{PSA}+1)$ velocity from −0.06 to 0.14 (first and third quartiles of the fitted velocities, respectively) corresponds to a 2.05 fold increase in the hazard of GR. In terms of the predictive performance, we found that the area under the receiver operating characteristic curves (Rizopoulos et al., 2017) was 0.61, 0.65, and 0.59 at years 1, 2, and 3 of follow‐up, respectively. Parameter estimates are presented in detail in Web Appendix C.

In PRIAS, the interval $l_{i}<T_{i}^{*}\leq r_{i}$ in which GR is detected depends on the PSA‐DT of the patient. However, because the parameters are estimated using a full likelihood approach (Tsiatis and Davidian, 2004), the joint model gives valid estimates for all of the parameters, under the condition that the model is correctly specified (see Web Appendix A.2 and C.3). To this end, we performed several sensitivity analysis in our model (e.g., changing the position of the knots, etc.) to investigate the fit of the model and also the robustness of the results. In all of our attempts, the same conclusions were reached, namely that the velocity of the longitudinal outcome is more strongly associated with the hazard of GR than the value.

### 5.2 Personalized Schedules for the First Demonstration Patient

**Figure 2:**
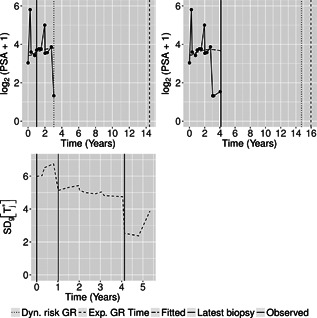
Top panel: fitted versus observed ${\text{log}}_{2}(\mathrm{PSA}+1)$ profile, history of repeat biopsies, and corresponding personalized schedules for the first demonstration patient. Bottom panel: history of repeat biopsies and standard deviation ${\mathrm{SD}}_{g}(T_{j}^{*})=\sqrt{{\mathrm{var}}_{g}(T_{j}^{*})}$ of the posterior predictive distribution of time of GR over time for the first demonstration patient.

We now demonstrate the functioning of the personalized schedules for the first demonstration patient (see Web Appendix D for the other two demonstration patients). The fitted and observed ${\text{log}}_{2}(\mathrm{PSA}+1)$ profile, time of latest biopsy, and proposed biopsy times *u* for him are shown in the top panel of Figure 2. We can see that with a consistently decreasing PSA and negative repeat biopsy between year 3 and 4.5, the proposed time of biopsy based on the dynamic risk of GR has increased from 3.05 years (κ=0.94) to 14.73 years (κ=0.96) in this period. The proposed time of biopsy based on expected time of GR has also increased from 14.40 to 15.97 years. We can also see in the bottom panel of Figure 2 that after each negative repeat biopsy, $\mathrm{SD}[T_{j}^{*}]=\sqrt{{\mathrm{var}}_{g}(T_{j}^{*})}$ decreases sharply. Thus, if the expected time of GR‐based approach is used, then the offset $O_{j}^{S}$ will be smaller on average for biopsies scheduled after the second repeat biopsy than those scheduled after the first repeat biopsy.

## 6 Simulation Study

In Section 5.2, we demonstrated that the personalized schedules, schedule future biopsies according to the historical data of each patient. However, we could not perform a full‐scale comparison between personalized and PRIAS schedules, because the true time of GR was not known for the PRIAS patients. To this end, we conducted a simulation study comparing personalized schedules with PRIAS and annual schedule, whose details are presented next.

### 6.1 Simulation Setup

The population of AS patients in this simulation study is assumed to have the same entrance criteria as that of PRIAS. The PSA and hazard of GR for these patients follow a joint model of the form postulated in Section 5.1, with the only change that ${\text{log}}_{2}\mathrm{PSA}$ levels are used as the outcome. The population joint model parameters are equal to the posterior mean of parameters estimated from the corresponding joint model fitted to the PRIAS dataset. We intend to test the efficacy of different schedules for a population which has patients with both faster as well as slowly‐progressing PCa. This rate of progression is not only manifested via PSA profiles but also via the baseline hazard. We assume that there are three equal sized subgroups $G_{1}$, $G_{2}$, and $G_{3}$ of patients in the population, each with a baseline hazard from a Weibull distribution, with the following shape and scale parameters (k,λ): (1.5,4), (3,5), and (4.5,6) for $G_{1},G_{2}$, and $G_{3}$, respectively. The effect of these parameters is that the mean GR time is lowest in $G_{1}$ (fast PCa progression) and highest in $G_{3}$ (slow PCa progression).

From this population, we have sampled 500 datasets with 1000 patients each. We generate a true GR time for each of the patients, and then sample a set of PSA measurements at the same time points as given in PRIAS protocol (see Web Appendix C). We then split the dataset into a training (750 patients) and a test (250 patients) part, and generate a random and non‐informative censoring time for the training patients. We next fit a joint model of the specification given in (7) and (8) to each of the 500 training datasets and obtain MCMC samples from the 500 sets of the posterior distribution of the parameters. Using these fitted joint models, we obtain the posterior predictive distribution of time of GR for each of the 500×250 test patients. This distribution is further used to create personalized biopsy schedules for the test patients. For every test patient we conduct hypothetical biopsies using the following six types of schedules (abbreviated names in parenthesis): personalized schedules based on expected time of GR (Exp. GR time) and median time of GR (Med. GR time), personalized schedules based on dynamic risk of GR (Dyn. risk GR), a hybrid approach between median time of GR and dynamic risk of GR (Hybrid), PRIAS schedule and the annual schedule. The biopsies are conducted as per the algorithm in Figure 1.

To compare the aforementioned schedules we require estimates of the various measures of efficacy described in Section 4. To this end, for schedule *S*, we compute pooled estimates of mean offset $E(O_{j}^{S})$ and variance of offset $\mathrm{var}(O_{j}^{S})$, as below (estimates for $N_{j}^{S}$ are similar):

$$\begin{array}{ccc} \hat{E(O_{j}^{S})} & = & \frac{\sum_{k=1}^{500}n_{k}\hat{E(O_{k}^{S})}}{\sum_{k=1}^{500}n_{k}},\\ \hat{\mathrm{var}(O_{j}^{S})} & = & \frac{\sum_{k=1}^{500}(n_{k}-1)\hat{\mathrm{var}(O_{k}^{S})}}{\sum_{k=1}^{500}(n_{k}-1)}, \end{array}$$

where $n_{k}$ denotes the number of test patients, $\hat{E(O_{k}^{S})}=\sum_{l=1}^{n_{k}}O_{\mathrm{kl}}^{S}/n_{k}$ is the estimated mean and $\hat{\mathrm{var}(O_{k}^{S})}=\sum_{l=1}^{n_{k}}{\left\{O_{\mathrm{kl}}^{S}-\hat{E(O_{k}^{S})}\right\}}^{2}/(n_{k}-1)$ is the estimated variance of the offset for the *k*‐th simulation. The offset for the *l*‐th test patient of the *k*‐th dataset is denoted by $O_{\mathrm{kl}}^{S}$.

### 6.2 Results

**Figure 3:**
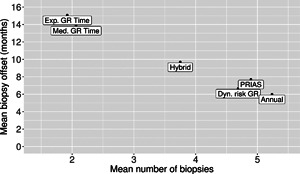
Estimated mean number of biopsies conducted until Gleason reclassification (GR) is detected, and mean offset (difference in time at which GR is detected and the true time of GR, in months) for the simulated (500 datasets) test patients, across different schedules. Types of personalized schedules (full names in brackets): Exp. GR time (expected time of GR), Med. GR time (median time of GR), Dyn. risk GR (schedules based on dynamic risk of GR), hybrid (a hybrid approach between median time of GR and dynamic risk of GR). Annual corresponds to a schedule of yearly biopsies and PRIAS corresponds to biopsies as per PRIAS protocol.

**Figure 4:**
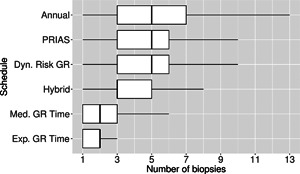
Boxplot showing variation in number of biopsies conducted by various biopsy schedules for the simulated (500 datasets) test patients. Biopsies are conducted until Gleason reclassification (GR) is detected. Types of personalized schedules (full names in brackets): Exp. GR Time (expected time of GR), Med. GR time (median time of GR), Dyn. risk GR (schedules based on dynamic risk of GR), hybrid (a hybrid approach between median time of GR and dynamic risk of GR). Annual corresponds to a schedule of yearly biopsies and PRIAS corresponds to biopsies as per PRIAS protocol.

**Figure 5:**
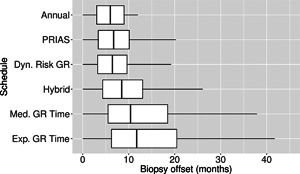
Boxplot showing variation in biopsy offset (difference in time at which Gleason reclassification, also known as GR, is detected and the true time of GR, in months) for the simulated (500 datasets) test patients, across different schedules. Types of personalized schedules (full names in brackets): Exp. GR time (expected time of GR), Med. GR time (median time of GR), Dyn. risk GR (schedules based on dynamic risk of GR), hybrid (a hybrid approach between median time of GR and dynamic risk of GR). Annual corresponds to a schedule of yearly biopsies and PRIAS corresponds to biopsies as per PRIAS protocol.

The pooled estimates of the aforementioned measures are summarized in Table 1. In addition, estimated values of $E(O_{j}^{S})$ are plotted against $E(N_{j}^{S})$ in Figure 3. The figure shows that across the schedules there is an inverse relationship between number $E(O_{j}^{S})$ and $E(N_{j}^{S})$. For example, the annual schedule conducts on average 5.2 biopsies to detect GR, which is the highest among all schedules. However, it has the least average offset of 6 months as well. On the other hand, the schedule based on expected time of GR conducts only 1.9 biopsies on average to detect GR, the least among all schedules, but it also has the highest average offset of 15 months (similar for median time of GR). Since the annual schedule attempts to contain the offset within a year it has the least $\mathrm{SD}(O_{j}^{S})=\sqrt{\mathrm{var}(O_{j}^{S})}$ (Figure 5). However, to achieve this, it conducts a wide range of number of biopsies from patient to patient, i.e., highest $\mathrm{SD}(N_{j}^{S})=\sqrt{\mathrm{var}(N_{j}^{S})}$ (Figure 4). In this regard, schedules based on expected and median time of GR perform the opposite of annual schedule.

**Table 1:** Estimated mean and standard deviation (SD), of the number of biopsies $N_{j}^{S}$ conducted until Gleason reclassification (GR) is detected, and of the offset $O_{j}^{S}$ (difference in time at which GR is detected and the true time of GR, in months), for the simulated (500 datasets) test patients, across different schedules and subgroups. Patients in subgroup $G_{1}$ have the fastest prostate cancer progression rate, whereas patients in subgroup $G_{3}$ have the slowest progression rate. Types of personalized schedules (full names in brackets): Exp. GR time (expected time of GR), Med. GR time (median time of GR), Dyn. risk GR (schedules based on dynamic risk of GR), hybrid (a hybrid approach between median time of GR and dynamic risk of GR). Annual corresponds to a schedule of yearly biopsies and PRIAS corresponds to biopsies as per PRIAS protocol

a) All hypothetical subgroups				
Schedule	$E(N_{j}^{S})$	$E(O_{j}^{S})$	$\mathrm{SD}(N_{j}^{S})$	$\mathrm{SD}(O_{j}^{S})$
Annual	5.24	6.01	2.53	3.46
PRIAS	4.90	7.71	2.36	6.31
Dyn. risk GR	4.69	6.66	2.19	4.38
Hybrid	3.75	9.70	1.71	7.25
Med. GR time	2.06	13.88	1.41	11.80
Exp. GR time	1.92	15.08	1.19	12.11
Hypothetical subgroup $G_{1}$				
Schedule	$E(N_{j}^{S})$	$E(O_{j}^{S})$	$\mathrm{SD}(N_{j}^{S})$	$\mathrm{SD}(O_{j}^{S})$
Annual	4.32	6.02	3.13	3.44
PRIAS	4.07	7.44	2.88	6.11
Dyn. risk GR	3.85	6.75	2.69	4.44
Hybrid	3.25	10.25	2.16	8.07
Med. GR time	1.84	20.66	1.76	14.62
Exp. GR time	1.72	21.65	1.47	14.75
Hypothetical subgroup $G_{2}$				
Schedule	$E(N_{j}^{S})$	$E(O_{j}^{S})$	$\mathrm{SD}(N_{j}^{S})$	$\mathrm{SD}(O_{j}^{S})$
Annual	5.18	5.98	2.13	3.47
PRIAS	4.85	7.70	2.00	6.29
Dyn. risk GR	4.63	6.66	1.82	4.37
Hybrid	3.68	10.32	1.37	7.45
Med. GR time	1.89	12.33	1.16	9.44
Exp. GR time	1.77	13.54	0.98	9.83
Hypothetical subgroup $G_{3}$				
Schedule	$E(N_{j}^{S})$	$E(O_{j}^{S})$	$\mathrm{SD}(N_{j}^{S})$	$\mathrm{SD}(O_{j}^{S})$
Annual	6.20	6.02	1.76	3.46
PRIAS	5.76	7.98	1.71	6.51
Dyn. risk GR	5.58	6.58	1.56	4.33
Hybrid	4.32	8.55	1.26	5.91
Med. GR time	2.45	8.70	1.15	6.32
Exp. GR time	2.27	10.09	0.99	7.47

The PRIAS schedule conducts only 0.3 biopsies less than the annual schedule, but with a higher $\mathrm{SD}(O_{j}^{S})$, early detection is not always guaranteed. In comparison, the dynamic risk of GR‐based schedule performs slightly better than the PRIAS schedule in all four criteria. The hybrid approach combines the benefits of methods with low $E(N_{j}^{S})$ and $\mathrm{SD}(N_{j}^{S})$, and methods with low $E(O_{j}^{S})$ and $\mathrm{SD}(O_{j}^{S})$. It conducts 1.5 biopsies less than the annual schedule on average and with a $E(O_{j}^{S})$ of 9.7 months it detects GR within a year since its occurrence. Moreover, it has both $\mathrm{SD}(N_{j}^{S})$ and $\mathrm{SD}(O_{j}^{S})$ comparable to PRIAS.

The performance of each schedule differs for the three subgroups $G_{1},G_{2}$, and $G_{3}$. The annual schedule remains the most consistent across subgroups in terms of the offset, but it conducts two extra biopsies for the subgroup $G_{3}$ (slowly‐progressing PCa) than $G_{1}$ (faster‐progressing PCa). The performance of schedule based on expected time of GR is the most consistent in terms of the number of biopsies but it detects GR a year later on average in subgroup $G_{1}$ than $G_{3}$. For the dynamic risk of GR‐based schedule and the hybrid schedule, the dynamics are similar to that of the annual schedule. Unlike the latter two schedules, the PRIAS schedule not only conducts more biopsies in $G_{3}$ than $G_{1}$ but also detects GR later in $G_{3}$ than $G_{1}$.

The choice of a suitable schedule using (5) depends on the chosen measure for evaluation of schedules. In this regard, the schedules we compared either have high $\mathrm{SD}(O_{j}^{S})$ and low $\mathrm{SD}(N_{j}^{S})$, or vice versa (Table 1). Thus, applying a cutoff on $E(O_{j}^{S})$ when $\mathrm{SD}(O_{j}^{S})$ is high may not be as fruitful (same for $N_{j}^{S}$) as applying a cutoff on $\mathrm{SD}(O_{j}^{S})$ or quantile(s) of $O_{j}^{S}$. For example, the schedule based on the dynamic risk of GR is suitable if on average the least number of biopsies are to be conducted to detect GR, while simultaneously making sure that at least 90% of the patients have an average offset less than 1 year.

## 7 Discussion

In this article, we presented personalized schedules based on joint models for time‐to‐event and longitudinal data, for surveillance of PCa patients. These schedules are dynamic in nature, and at any given follow‐up time, utilize a patient's historical PSA measurements and repeat biopsies conducted up to that time. We proposed two types of personalized schedules, namely those based on expected and median time of GR of a patient, and those based on the dynamic risk of GR. We also proposed a combination (hybrid approach) of these two approaches, which is useful in scenarios where the variance of time of GR for a patient is high. We then proposed criteria for evaluation of various schedules and a method to select a suitable schedule.

We demonstrated the dynamic and personalized nature of our schedules using the PRIAS dataset. We observed that a recent biopsy impacts the schedules more than recent PSA measurements, which correlates with biopsies being more reliable. Since true GR time is not known for PRIAS patients, we conducted a simulation study to compare personalized schedules with PRIAS and annual schedules. The latter two schedules are already in practice. Hence, it can be argued that the maximum possible offsets due to these schedules (1 and 3 years, respectively) are acceptable to doctors. Thus, less frequent schedules with offset under 1 year may reduce the burden of biopsies while simultaneously being practical. For example, for slowly‐progressing patients in our simulation study, we observed that the schedule based on expected time of GR conducts on average two biopsies and has an average offset of 10 months. In comparison, annual schedule conducts six biopsies on average and gives an offset smaller by only 4 months, making the personalized schedule a suitable alternative. For high‐risk patients, however, early detection (annual or PRIAS schedule) may be necessary, given the rapidness of progression. When it is not known in advance if a patient will have a fast or slow‐progression of PCa, the hybrid approach may be used. It conducts one biopsy less than the annual schedule in faster‐progressing PCa patients and has an average offset of 10.25 months. For slowly‐progressing PCa patients it conducts two biopsies less than the annual schedule and has an average offset of 8.55 months.

More personalized schedules can be added to the current set, using loss functions which asymmetrically penalize overshooting/undershooting the target GR time. For dynamic risk of GR‐based schedules, more simulations are required to compare data‐driven κ values (e.g., $F_{1}$ score), with κ chosen using decision analytic approaches such as the net benefit measure (Vickers and Elkin, 2006), and with various fixed κ values used by doctors in practice. In general, the Gleason scores are susceptible to inter‐observer variation (Carlson et al., 1998). Schedules which account for error in the measurement of time of GR will be interesting to investigate further (Coley et al., 2017). Lastly, there is potential for including diagnostic information from magnetic resonance imaging (MRI) or DRE. When such information is not continuous in nature, our proposed methodology can be easily extended by utilizing the framework of generalized linear mixed models.

## 8 Supplementary Materials

Web Appendix A, B, and C, D referenced in Sections 2, 3.3, and 5, respectively, and the R code for fitting the joint model to the PRIAS dataset, and for the simulation study are available with this article at the *Biometrics* website on Wiley Online Library.

## Supporting information

Supplementary Data S1.

Supplementary Data Code S1.
