# Supplementary material for: Personalized schedules for surveillance of low‐risk prostate cancer patients
Source: Biometrics. 2018 Jul 23;75(1):153–62. doi: 10.1111/biom.12940 (PMC7380003; doi:10.1111/biom.12940)
Supplement: Supplementary file 1 — Supplementary Data S1. [file BIOM-75-153-s001.pdf]

BIOMETRICS 00, 1–30 DOI: 10.1111/j.1541-0420.2005.00454.x
December 0000

Web-based Supplementary Materials for "Personalized Schedules for Surveillance of Low-Risk Prostate Cancer Patients"

Anirudh Tomer[1,*], Daan Nieboer[2], Monique J. Roobol[3], Ewout W. Steyerberg[2,4], and Dimitris Rizopoulos[1]

[1]Department of Biostatistics, Erasmus University Medical Center, the Netherlands
[2]Department of Public Health, Erasmus University Medical Center, the Netherlands
[3]Department of Urology, Erasmus University Medical Center, the Netherlands
[4]Department of Medical Statistics and Bioinformatics, Leiden University Medical Center, the Netherlands
*email: a.tomer@erasmusmc.nl

This paper has been submitted for consideration for publication in *Biometrics*

Web Appendix A. Joint Model for Time-to-Event and Longitudinal Outcomes

We start with a short introduction of the joint modeling framework we will use in our following developments. Let T_i^* denote the true Gleason reclassification (GR) time for the i-th patient and let S be the schedule of his biopsies. Let the vector of the time of biopsies be denoted by $T_i^S = \{T_{i0}^S, T_{i1}^S, \ldots, T_{iN_i^S}^S; T_{ij}^S < T_{ik}^S, \forall j < k\}$, where N_i^S are the total number of biopsies conducted. Because biopsy schedules are periodical, T_i^* cannot be observed directly and it is only known to fall in an interval $l_i < T_i^* \leqslant r_i$, where $l_i = T_{iN_i^S - 1}^S, r_i = T_{iN_i^S}^S$ if GR is observed, and $l_i = T_{iN_i^S}^S, r_i = \infty$ if GR is not observed yet. Further let $\boldsymbol{y}_i$ denote the $n_i \times 1$ vector of prostate-specific antigen (PSA) levels for the i-th patient. For a sample of n patients the observed data is denoted by $\mathcal{D}_n = \{l_i, r_i, \boldsymbol{y}_i; i = 1, \ldots, n\}$.

The longitudinal outcome of interest, namely PSA level, is continuous in nature and thus to model it the joint model utilizes a linear mixed effects model (LMM) of the form:

$$\begin{aligned} y_i(t) &= m_i(t) + \varepsilon_i(t) \\ &= \boldsymbol{x}_i^T(t)\boldsymbol{\beta} + \boldsymbol{z}_i^T(t)\boldsymbol{b}_i + \varepsilon_i(t), \end{aligned}$$

where $\boldsymbol{x}_i(t)$ and $\boldsymbol{z}_i(t)$ denote the row vectors of the design matrix for fixed and random effects, respectively. The fixed and random effects are denoted by $\boldsymbol{\beta}$ and $\boldsymbol{b}_i$, respectively. The random effects are assumed to be normally distributed with mean zero and $q \times q$ covariance matrix $\boldsymbol{D}$. The true and unobserved, error free PSA level at time t is denoted by $m_i(t)$. The error $\varepsilon_i(t)$ is assumed to be t-distributed with three degrees of freedom and scale σ (see **??**), and is independent of the random effects $\boldsymbol{b}_i$.

To model the effect of PSA on hazard of GR, joint models utilize a relative risk sub-model. The hazard of GR for patient i at any time point t, denoted by $h_i(t)$, depends on a function of subject specific linear predictor $m_i(t)$ and/or the random effects:

$$\begin{aligned} h_i(t \mid \mathcal{M}_i(t), \boldsymbol{w}_i) &= \lim_{\Delta t \to 0} \frac{\Pr\big\{t \leqslant T_i^* < t + \Delta t \mid T_i^* \geqslant t, \mathcal{M}_i(t), \boldsymbol{w}_i\big\}}{\Delta t} \\ &= h_0(t) \exp\big[\boldsymbol{\gamma}^T \boldsymbol{w}_i + f\{\mathcal{M}_i(t), \boldsymbol{b}_i, \boldsymbol{\alpha}\}\big], \quad t > 0, \end{aligned}$$

where $\mathcal{M}_i(t) = \{m_i(v), 0 \leqslant v \leqslant t\}$ denotes the history of the underlying PSA levels up to time t. The vector of baseline covariates is denoted by $\boldsymbol{w}_i$, and $\boldsymbol{\gamma}$ are the corresponding parameters. The function $f(\cdot)$ parametrized by vector $\boldsymbol{\alpha}$ specifies the functional form of PSA levels (Brown, 2009; Rizopoulos, 2012; Taylor et al., 2013; Rizopoulos et al., 2014) that is used in the linear predictor of the relative risk sub-model. Some functional forms relevant to the problem at hand are the following:

$$\begin{cases} f\{\mathcal{M}_i(t), \boldsymbol{b}_i, \boldsymbol{\alpha}\} = \alpha m_i(t), \\ f\{\mathcal{M}_i(t), \boldsymbol{b}_i, \boldsymbol{\alpha}\} = \alpha_1 m_i(t) + \alpha_2 m_i'(t), \quad \text{with } m_i'(t) = \frac{\mathrm{d}m_i(t)}{\mathrm{d}t}. \end{cases}$$

These formulations of $f(\cdot)$ postulate that the hazard of GR at time t may be associated with the underlying level $m_i(t)$ of the PSA at t, or with both the level and velocity $m_i'(t)$ of the PSA at t. Lastly, $h_0(t)$ is the baseline hazard at time t, and is modeled flexibly using P-splines. More specifically:

$$\log h_0(t) = \gamma_{h_0,0} + \sum_{q=1}^{Q} \gamma_{h_0,q} B_q(t, \boldsymbol{v}),$$

where $B_q(t, \boldsymbol{v})$ denotes the q-th basis function of a B-spline with knots $\boldsymbol{v} = v_1, \ldots, v_Q$ and vector of spline coefficients γ_{h_0}. To avoid choosing the number and position of knots in the spline, a relatively high number of knots (e.g., 15 to 20) are chosen and the corresponding B-spline regression coefficients γ_{h_0} are penalized using a differences penalty (Eilers and Marx, 1996).

Web Appendix A.1 *Parameter Estimation*

We estimate parameters of the joint model using Markov chain Monte Carlo (MCMC) methods under the Bayesian framework. Let $\boldsymbol{\theta}$ denote the vector of the parameters of the joint model. The joint model postulates that given the random effects, time to GR and longitudinal responses taken over time are all mutually independent. Under this assumption the posterior distribution of the parameters is given by:

$$\begin{aligned}
p(\boldsymbol{\theta}, \boldsymbol{b} \mid \mathcal{D}_n) &\propto \prod_{i=1}^{n} p(l_i, r_i, \boldsymbol{y}_i \mid \boldsymbol{b}_i, \boldsymbol{\theta}) p(\boldsymbol{b}_i \mid \boldsymbol{\theta}) p(\boldsymbol{\theta}) \\
&\propto \prod_{i=1}^{n} p(l_i, r_i \mid \boldsymbol{b}_i, \boldsymbol{\theta}) p(\boldsymbol{y}_i \mid \boldsymbol{b}_i, \boldsymbol{\theta}) p(\boldsymbol{b}_i \mid \boldsymbol{\theta}) p(\boldsymbol{\theta}), \\
p(\boldsymbol{b}_i \mid \boldsymbol{\theta}) &= \frac{1}{\sqrt{(2\pi)^q \det(\boldsymbol{D})}} \exp(\boldsymbol{b}_i^T \boldsymbol{D}^{-1} \boldsymbol{b}_i),
\end{aligned}$$

where the likelihood contribution of longitudinal outcome conditional on random effects is:

$$\begin{aligned}
p(\boldsymbol{y}_i \mid \boldsymbol{b}_i, \boldsymbol{\theta}) &= \frac{1}{\left(\sqrt{2\pi\sigma^2}\right)^{n_i}} \exp\left(- \frac{\lVert \boldsymbol{y}_i - \boldsymbol{X}_i \boldsymbol{\beta} - \boldsymbol{Z}_i \boldsymbol{b}_i \rVert^2}{\sigma^2} \right), \\
\boldsymbol{X}_i &= \{\boldsymbol{x}_i(t_{i1})^T, \ldots, \boldsymbol{x}_i(t_{in_i})^T\}^T, \\
\boldsymbol{Z}_i &= \{\boldsymbol{z}_i(t_{i1})^T, \ldots, \boldsymbol{z}_i(t_{in_i})^T\}^T.
\end{aligned}$$

The likelihood contribution of the time to GR outcome is given by:

$$p(l_i, r_i \mid \boldsymbol{b}_i, \boldsymbol{\theta}) = \exp\Big\{ - \int_0^{l_i} h_i(s \mid \mathcal{M}_i(s), \boldsymbol{w}_i) \mathrm{d}s \Big\} - \exp\Big\{ - \int_0^{r_i} h_i(s \mid \mathcal{M}_i(s), \boldsymbol{w}_i) \mathrm{d}s \Big\}. \quad (1)$$

The integral in (1) does not have a closed-form solution, and therefore we use a 15-point Gauss-Kronrod quadrature rule to approximate it.

We use independent normal priors with zero mean and variance 100 for the fixed effects $\boldsymbol{\beta}$, and inverse Gamma prior with shape and rate both equal to 0.01 for the parameter σ^2. For the variance-covariance matrix $\boldsymbol{D}$ of the random effects we take inverse Wishart prior with an identity scale matrix and degrees of freedom equal to q (number of random effects). For the relative risk model's parameters $\boldsymbol{\gamma}$ and the association parameters $\boldsymbol{\alpha}$, we use independent normal priors with zero mean and variance 100.

Web Appendix A.2 *Interval Censoring in Time of Gleason Reclassification*

The true time of GR T_i^* is not known for any of the patients. In order to detect GR, PRIAS uses a fixed schedule of biopsies wherein biopsies are conducted at year one, year four, year seven and year ten of follow-up, and every five years thereafter. However, PRIAS switches to a more frequent annual biopsy schedule for faster-progressing patients. These are patients with PSA doubling time (PSA-DT) between 0 and 10 years, which is measured as the inverse of the slope of the regression line through the base two logarithm of PSA values. Thus, the interval $l_i < T_i^* \leqslant r_i$ in which GR is detected depends on the observed PSA values.

It is natural to question in this scenario if the parameters of the joint model are affected by PSA-DT dependent interval censoring. However, because the parameters of the joint model are estimated using a full likelihood approach (Tsiatis and Davidian, 2004), the joint model allows the schedule of biopsies to depend upon the observed PSA values (e.g., via PSA-DT), under the condition that the model is correctly specified (we discuss this aspect in Web Appendix C). To show this, consider the following full general specification of the joint model that we use. Let $\boldsymbol{y}_i$ denote the observed PSA measurements for the i-th patient, and l_i, r_i denote the two time points of the interval in which GR occurs for the i-th patient. In addition let T_i^S and $\mathcal{V}_i$ denote the schedule of biopsies and schedule of PSA measurements, respectively. Under the assumption that both of these schedules may depend upon only the observed $\boldsymbol{y}_i$, the joint likelihood of all four processes is given by:

$$p(\boldsymbol{y}_i, l_i, r_i, T_i^S, \mathcal{V}_i \mid \boldsymbol{\theta}, \boldsymbol{\psi}) = p(\boldsymbol{y}_i, l_i, r_i \mid \theta) \times p(T_i^S, \mathcal{V}_i \mid \boldsymbol{y}_i, \boldsymbol{\psi}). \tag{2}$$

From this decomposition we can see that even if the processes T_i^S and $\mathcal{V}_i$ may be determined from $\boldsymbol{y}_i$, if we are interested in the parameters $\boldsymbol{\theta}$ of the joint distribution of longitudinal and event outcome, we can maximize the likelihood based on the first term and ignore the second term. In other words, the second term will not carry information for $\boldsymbol{\theta}$.

It is important to note that, since we use a full likelihood approach with an interval

censoring specification, the estimates that we obtain are consistent and asymptotically unbiased (Gentleman and Geyer, 1994), despite the interval censoring observed.

Web Appendix B. Derivations and Computations for $E_g(T_j^*)$ and $\mathrm{var}_g(T_j^*)$

In this section we present the derivations for $E_g(T_j^*)$ and $\mathrm{var}_g(T_j^*)$, corresponding to Equations 3 and 4 of the main manuscript, respectively. To this end, we first expand the formula for dynamic survival probability presented in Section 3.2 of the main manuscript.

$$\begin{aligned}\pi_j(u \mid t, s) &= \Pr\big\{T_j^* \geqslant u \mid T_j^* > t, \mathcal{Y}_j(s), D_n\big)\\ &= \int\int \Pr\big(T_j^* \geqslant u \mid T_j^* > t, \boldsymbol{b}_j, \boldsymbol{\theta}\big) p\big\{\boldsymbol{b}_j \mid T_j^* > t, \mathcal{Y}_j(s), \boldsymbol{\theta}\big\} p(\boldsymbol{\theta} \mid \mathcal{D}_n) \mathrm{d}\boldsymbol{b}_j \mathrm{d}\boldsymbol{\theta} \\ &= \int\int \frac{\exp\big\{-H_j(u|\boldsymbol{b}_j, \boldsymbol{\theta})\big\}}{\exp\big\{-H_j(t|\boldsymbol{b}_j, \boldsymbol{\theta})\big\}} p\big\{\boldsymbol{b}_j \mid T_j^* > t, \mathcal{Y}_j(s), \boldsymbol{\theta}\big\} p(\boldsymbol{\theta} \mid \mathcal{D}_n) \mathrm{d}\boldsymbol{b}_j \mathrm{d}\boldsymbol{\theta},\end{aligned} \tag{3}$$

where $H_j(u|\boldsymbol{b}_j, \boldsymbol{\theta}) = \int_0^u h_i(s \mid \boldsymbol{b}_j, \boldsymbol{\theta}\big)\mathrm{d}s$ is the cumulative hazard up to time point u.

Web Appendix B.1 *Derivation of $E_g(T_j^*)$, Shown in Equation 3 of the Main Manuscript*

$$E_g(T_j^*) = \int_t^\infty T_j^* g(T_j^*) \mathrm{d}T_j^*.$$

Using integration by parts, wherein $\mathrm{d}\big\{-\pi_j(T_j^* \mid t, s)\big\}/\mathrm{d}T_j^* = g(T_j^*)$,

$$\begin{aligned}E_g(T_j^*) &= \Big[-T_j^* \pi_j(T_j^* \mid t, s)\Big]_t^\infty + \int_t^\infty \pi_j(T_j^* \mid t, s) \frac{\mathrm{d}(T_j^*)}{\mathrm{d}T_j^*} \mathrm{d}T_j^* \\ &= t\pi_j(t \mid t, s) - \lim_{T_j^* \to \infty} T_j^* \pi_j(T_j^* \mid t, s) \\ &\quad + \int_t^\infty \pi_j(T_j^* \mid t, s) \mathrm{d}T_j^*,\end{aligned}$$

where $\pi_j(t \mid t, s) = \Pr\big\{T_j^* \geqslant t \mid T_j^* > t, \mathcal{Y}_j(s), D_n\big\} = 1$. As for $\lim_{T_j^* \to \infty} T_j^* \pi_j(T_j^* \mid t, s)$, the limit can be interchanged with the integral in Equation 3, because as $T_j^* \to \infty$ the integrand in the equation converges uniformly on the domain of $\big(\boldsymbol{b}_j, \boldsymbol{\theta}\big)$. Thus,

$$\begin{aligned}\lim_{T_j^* \to \infty} T_j^* \pi_j(T_j^* \mid t, s) = \int\int & \lim_{T_j^* \to \infty} \frac{T_j^*}{\exp\big\{H_j(T_j^* | \boldsymbol{b}_j, \boldsymbol{\theta})\big\}} \\ & \times \frac{p\big\{\boldsymbol{b}_j \mid T_j^* > t, \mathcal{Y}_j(s), \boldsymbol{\theta}\big\} p(\boldsymbol{\theta} \mid \mathcal{D}_n)}{\exp\big\{-H_j(t|\boldsymbol{b}_j, \boldsymbol{\theta})\big\}} \mathrm{d}\boldsymbol{b}_j \mathrm{d}\boldsymbol{\theta}.\end{aligned}$$

Using L'Hospital's rule,

$$\lim_{T_j^* \to \infty} T_j^* \pi_j(T_j^* \mid t, s) = \int\int \frac{1}{\lim_{T_j^* \to \infty} \exp\big\{H_j(T_j^* | \boldsymbol{b}_j, \boldsymbol{\theta})\big\} H_j'(T_j^* | \boldsymbol{b}_j, \boldsymbol{\theta})} \times \frac{p\big\{\boldsymbol{b}_j \mid T_j^* > t, \mathcal{Y}_j(s), \boldsymbol{\theta}\big\} p(\boldsymbol{\theta} \mid \mathcal{D}_n)}{\exp\big\{-H_j(t | \boldsymbol{b}_j, \boldsymbol{\theta})\big\}} \mathrm{d}\boldsymbol{b}_j \mathrm{d}\boldsymbol{\theta}$$

$$= \int\int 0 \times \frac{p\big\{\boldsymbol{b}_j \mid T_j^* > t, \mathcal{Y}_j(s), \boldsymbol{\theta}\big\} p(\boldsymbol{\theta} \mid \mathcal{D}_n)}{\exp\big\{-H_j(t | \boldsymbol{b}_j, \boldsymbol{\theta})\big\}} \mathrm{d}\boldsymbol{b}_j \mathrm{d}\boldsymbol{\theta}$$

$$= 0.$$

In light of these results, we obtain:

$$E_g(T_j^*) = t + \int_t^\infty \pi_j(T_j^* \mid t, s) \mathrm{d}T_j^*.$$

Web Appendix B.2 *Derivation of* $var_g(T_j^*)$*, Shown in Equation 4 of the Main Manuscript*

Since $\mathrm{var}_g(T_j^*) = E_g\{(T_j^*)^2\} - E_g(T_j^*)^2$, we first show the derivation for $E_g\{(T_j^*)^2\}$.

$$E_g\{(T_j^*)^2\} = \int_t^\infty (T_j^*)^2 g(T_j^*) \mathrm{d}T_j^*.$$

Using integration by parts, wherein $\mathrm{d}\big\{-\pi_j(T_j^* \mid t, s)\big\}/\mathrm{d}T_j^* = g(T_j^*)$,

$$E_g\big\{(T_j^*)^2\big\} = \Big[-(T_j^*)^2 \pi_j(T_j^* \mid t, s)\Big]_t^\infty + \int_t^\infty \pi_j(T_j^* \mid t, s) \frac{\mathrm{d}(T_j^*)^2}{\mathrm{d}T_j^*} \mathrm{d}T_j^*$$

$$= t^2 \pi_j(t \mid t, s) - \lim_{T_j^* \to \infty} (T_j^*)^2 \pi_j(T_j^* \mid t, s) + 2\int_t^\infty T_j^* \pi_j(T_j^* \mid t, s) \mathrm{d}T_j^*$$

$$= t^2 + 2\int_t^\infty T_j^* \pi_j(T_j^* \mid t, s) \mathrm{d}T_j^*.$$

Therefore,

$$\mathrm{var}_g(T_j^*) = t^2 + 2\int_t^\infty T_j^* \pi_j(T_j^* \mid t, s) \mathrm{d}T_j^* - \left[t^2 + \left\{\int_t^\infty \pi_j(T_j^* \mid t, s) \mathrm{d}T_j^*\right\}^2 + 2t\int_t^\infty \pi_j(T_j^* \mid t, s) \mathrm{d}T_j^*\right]$$

$$= 2\int_t^\infty (T_j^* - t) \pi_j(T_j^* \mid t, s) \mathrm{d}T_j^* - \left\{\int_t^\infty \pi_j(T_j^* \mid t, s) \mathrm{d}T_j^*\right\}^2.$$

Web Appendix B.3 *Computations for* $E_g(T_j^*)$ *and* $var_g(T_j^*)$

As we have shown above, we compute $E_g(T_j^*)$ and $\mathrm{var}_g(T_j^*)$ by first expressing them in terms of the dynamic survival probability in (3) and then calculating the survival probability

values. We preferred this approach over Monte Carlo methods to estimate $E_g(T_j^*)$ from $g(T_j^*)$, because sampling directly from $g(T_j^*)$ involved an additional step of sampling from the distribution $p(T_j^* \mid T_j^* > t, \boldsymbol{b}_j, \boldsymbol{\theta})$, as compared to the estimation of $\pi_j(u \mid t, s)$ (Rizopoulos, 2011). The former approach was thus computationally faster.

Web Appendix C. Fitting the Joint Model to the PRIAS Dataset

For each of the PRIAS patients, we know their age at the time of inclusion in AS, PSA history and the time interval in which GR is detected. PSA was measured at every three months for the first two years and every six months thereafter. For the longitudinal analysis of PSA we use $\log_2(\text{PSA}+1)$ measurements instead of the raw data (Lin et al., 2000; Pearson et al., 1994). The longitudinal sub-model of the joint model we fit is given by:

$$\begin{aligned}\log_2(\text{PSA}_i + 1)(t) &= \beta_0 + \beta_1(\text{Age}_i - 70) + \beta_2(\text{Age}_i - 70)^2 + \sum_{k=1}^{4} \beta_{k+2} B_k(t, \mathcal{K}) \\ &\quad + b_{i0} + b_{i1} B_7(t, 0.1) + b_{i2} B_8(t, 0.1) + \varepsilon_i(t),\end{aligned} \tag{4}$$

where $B_k(t, \mathcal{K})$ denotes the k-th basis function of a B-spline with three internal knots at $\mathcal{K} = \{0.1, 0.5, 4\}$ years, and boundary knots at zero and seven (0.99 quantile of the observed follow-up times) years. The spline for the random effects consists of one internal knot at 0.1 years and boundary knots at zero and seven years. Age of patients was median centered to avoid numerical instabilities during parameter estimation. The error $\varepsilon_i(t)$ is assumed to be t-distributed with three degrees of freedom and scale σ, and is independent of the random effects $\boldsymbol{b}_i$. For the relative risk sub-model the hazard function we fit is given by:

$$h_i(t) = h_0(t) \exp\bigl\{\gamma_1(\text{Age}_i - 70) + \gamma_2(\text{Age}_i - 70)^2 + \alpha_1 m_i(t) + \alpha_2 m'_i(t)\bigr\}, \tag{5}$$

where α_1 and α_2 are measures of strength of the association between hazard of GR and $\log_2(\text{PSA}_i + 1)$ value $m_i(t)$ and $\log_2(\text{PSA}_i + 1)$ velocity $m'_i(t)$, respectively.

Web Appendix C.1 *Parameter Estimates*

The posterior parameter estimates for the joint model we fitted to the PRIAS dataset are shown in Web Table 1 (longitudinal sub-model) and Web Table 2 (relative risk sub-model), and parameter estimates for the variance-covariance matrix from the longitudinal sub-model are the following:

$$\boldsymbol{D} = \begin{bmatrix} 0.268 & 0.055 & -0.043 \\ 0.055 & 0.712 & 0.257 \\ -0.043 & 0.257 & 0.591 \end{bmatrix}$$

For longitudinal sub-model parameter estimates, in Web Table 1 we can see that the age of the patient trivially affects the baseline $\log_2(\text{PSA}+1)$ values. Since the longitudinal evolution of $\log_2(\text{PSA} + 1)$ is modeled with non-linear terms, the interpretation of the coefficients corresponding to time is not straightforward. In lieu of the interpretation, in Web Figure 1 we present the fitted marginal evolution of $\log_2(\text{PSA} + 1)$ over a period of 10 years for a hypothetical patient who is included in AS at the age of 70 years. In addition we present plots of observed versus fitted profiles for nine randomly selected patients in Web Figure 3. Lastly, the quantile-quantile plot of subject-specific residuals in Figure 2 shows that the assumption of t-distributed (df=3) errors is reasonably met by the fitted model.

Web Table 1

Estimated mean and 95% credible interval for the parameters from the longitudinal sub-model of the joint model fitted to the PRIAS dataset.

Variable	Mean	Std. Dev	2.5%	97.5%	P
Intercept	2.686	0.009	2.668	2.704	<0.000
(Age – 70)	0.003	0.001	0.001	0.005	0.002
$(\text{Age} - 70)^2$	-0.001	1.073×10^{-4}	-0.001	-2.963×10^{-4}	<0.000
Spline: visit time [0.0, 0.1] years	0.049	0.008	0.035	0.065	<0.000
Spline: visit time [0.1, 0.5] years	0.258	0.012	0.235	0.281	<0.000
Spline: visit time [0.5, 4.0] years	0.282	0.018	0.244	0.320	<0.000
Spline: visit time [4.0, 7.0] years	0.412	0.023	0.367	0.460	<0.000
σ	0.147	0.001	0.146	0.149	

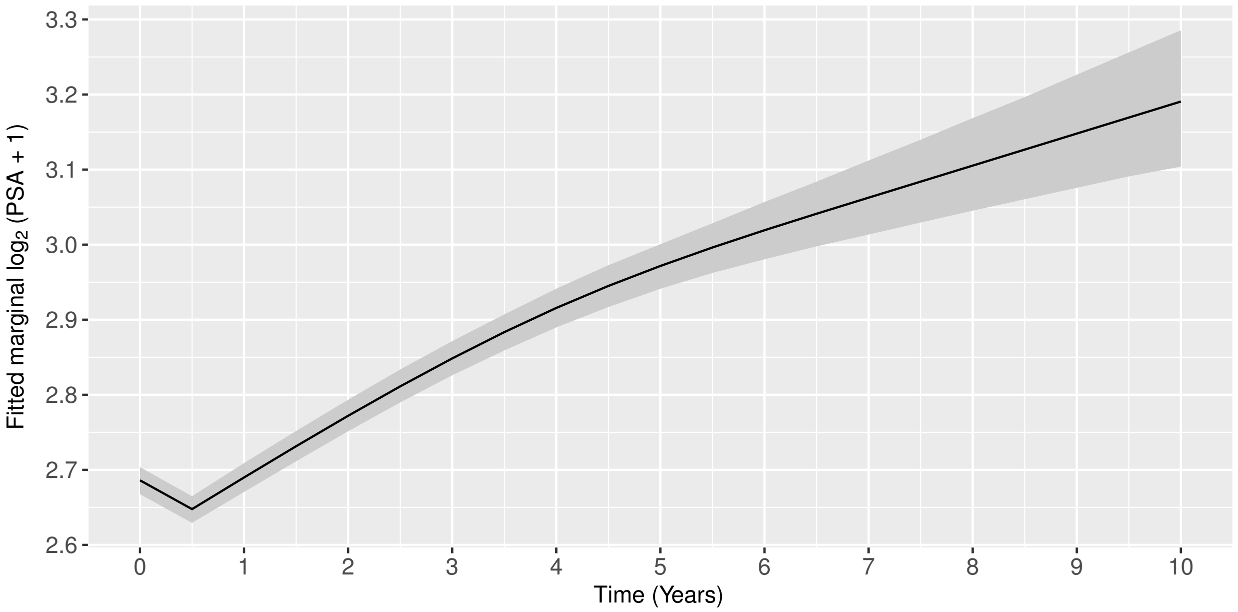

Web Figure 1. Fitted marginal evolution of $\log_2(\mathrm{PSA}+1)$ levels over a period of 10 years with 95% credible interval, for a hypothetical patient who is included in AS at the age of 70 years.

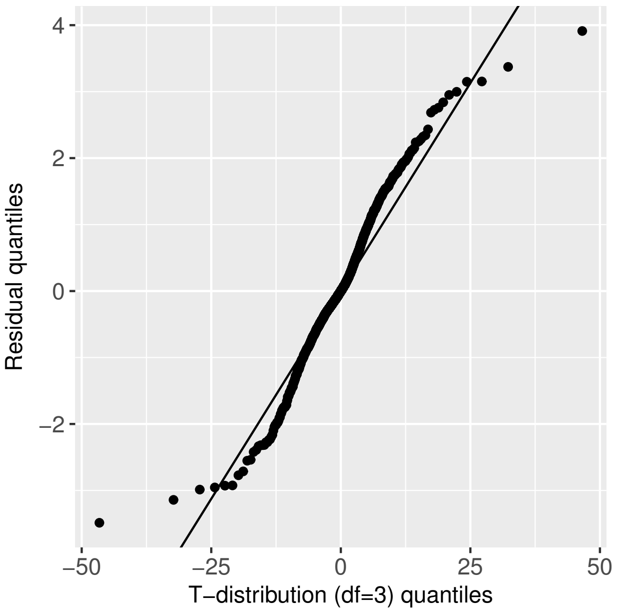

Web Figure 2. Quantile-quantile plot of subject-specific residuals from the joint model fitted to the PRIAS dataset.

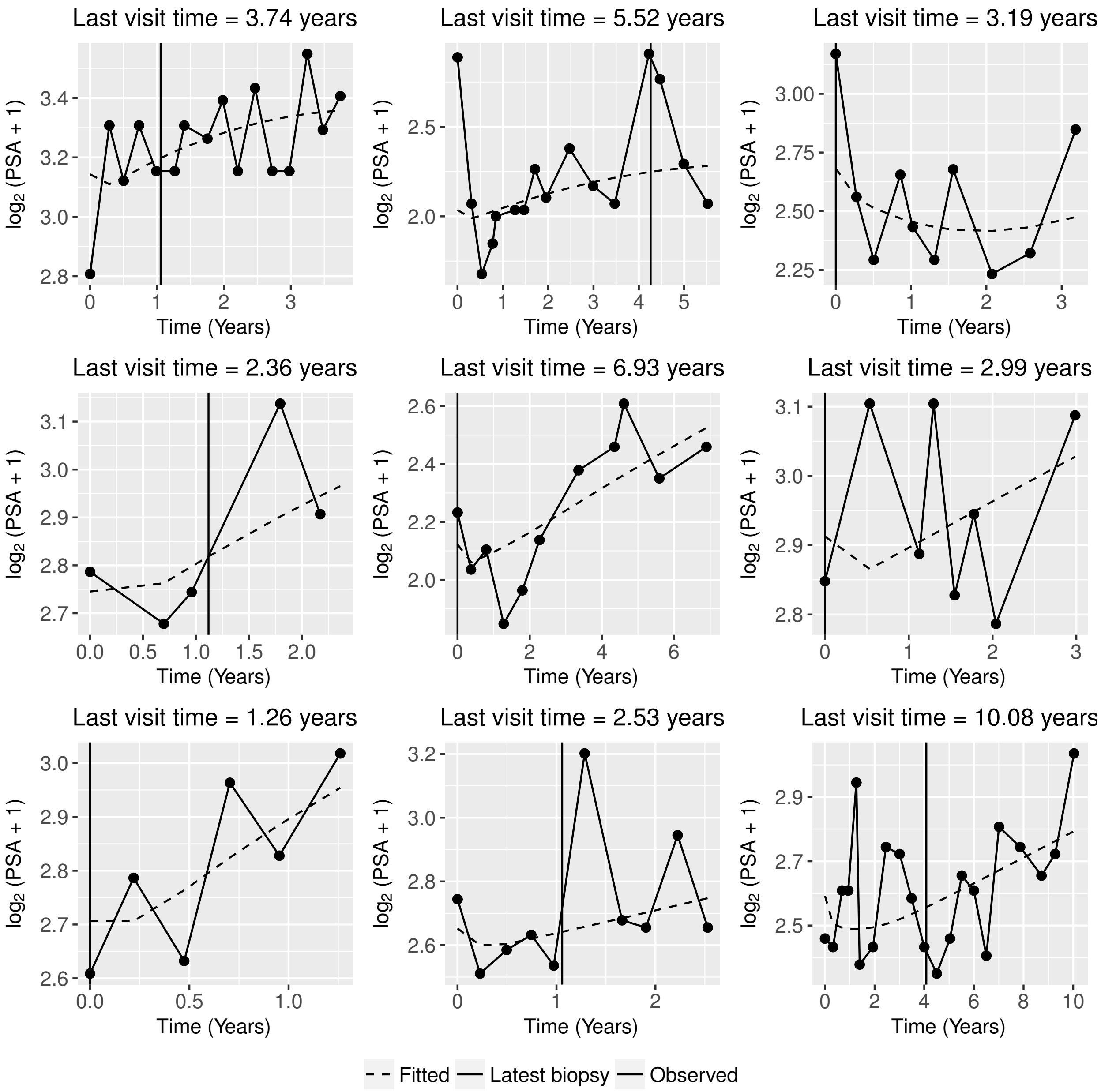

Web Figure 3. Fitted versus observed $\log_2(\mathrm{PSA}+1)$ profiles for nine randomly selected PRIAS patients. The fitted profiles utilize information from both the observed PSA levels and time of latest biopsy.

For the relative risk sub-model, the parameter estimates in Web Table 2 show that $\log_2(\text{PSA}+1)$ velocity and the age at the time of inclusion in AS are strongly associated with the hazard of GR. For any patient, an increase in $\log_2(\text{PSA}+1)$ velocity from -0.061 to 0.136 (first and third quartiles of the fitted velocities, respectively) corresponds to a 2.046 fold increase in the hazard of GR. An increase in age at the time of inclusion in AS from 65 years to 75 years (first and third quartiles of age in PRIAS dataset) corresponds to a 1.428 fold increase in the hazard of GR.

Web Table 2

Estimated mean and 95% credible interval for the parameters of the relative risk sub-model of the joint model fitted to the PRIAS dataset.

Variable	Mean	Std. Dev	2.5%	97.5%	P
$(\text{Age} - 70)$	0.036	0.006	0.024	0.047	<0.000
$(\text{Age} - 70)^2$	-0.001	0.001	-0.003	7.861×10^{-5}	0.084
$\log_2(\text{PSA}+1)$	-0.084	0.080	-0.241	0.072	0.296
$\text{Slope}(\log_2(\text{PSA}+1))$	3.580	0.403	2.815	4.373	<0.000

To compare the predictive performance of a models having association between hazard of GR and value of longitudinal outcome values, versus a model having the association with both value and velocity, we calculate the area under the receiver operating characteristic curves, also called AUC (Rizopoulos, Molenberghs, and Lesaffre, 2017), for these models (with the only change that $\log_2$ PSA levels are used as the outcome). Since in a joint model time dependent AUC is more relevant, we calculate the AUC at year one, year two and year three of follow-up in AS. The time window for which the AUC is calculated is one year. The resulting AUC are presented in Web Table 3.

Web Table 3

Area under the receiver operating characteristic curves (AUC), and 95% confidence interval in brackets. AUC's are calculated for two joint models: first one having association between hazard of GR and longitudinal outcome's value as well as velocity, and second one having association with only longitudinal outcome's value (with the only change that $\log_2$ *PSA levels are used as the outcome).*

Year	value and velocity association	value association
1	0.613 [0.582, 0.632]	0.595 [0.565, 0.618]
2	0.648 [0.608, 0.685]	0.609 [0.568, 0.654]
3	0.593 [0.560, 0.638]	0.590 [0.536, 0.628]

Web Appendix C.2 *PSA-DT Dependent Interval Censoring in Time of Gleason Reclassification*

In PRIAS, the interval $l_i < T_i^* \leqslant r_i$ in which GR is detected depends on the observed PSA values (via PSA-DT). It is natural to question in this scenario if the parameters of the joint model are affected by PSA-DT dependent interval censoring. To this end, we discussed via the formulation of the likelihood function in Web Appendix A.2, that the joint model gives consistent and asymptotically unbiased estimates of the parameters even if the interval censoring depends on PSA-DT, under the condition that the model is correctly specified. However, in this section we also demonstrate this via a simulated dataset of 750 patients. The true event times T_i^* for these patients were generated using parameters from a joint model fitted to the PRIAS dataset (with the only change that $\log_2$ PSA levels are used as the outcome). However this joint model did not include association between velocity of log PSA values and hazard of GR. That is, the hazard of GR $h_i(t)$ at any time t depends only on the underlying $\log_2$ PSA value $m_i(t)$ at that time. Furthermore, for these patients we used the schedule of PRIAS to generate the interval $l_i \leqslant T_i^* \leqslant r_i$ in which GR is detected. Thus the observed data for i-th patient is $\{\boldsymbol{y}_i, l_i, r_i\}$. Our aim is to show that if there is no association between $h_i(t)$ and velocity of log PSA value $m_i'(t)$, then even though the biopsy schedule depends on PSA-DT (which is a crude measure of PSA velocity), a joint model fitted with both value and velocity associations will have an insignificant velocity association. In the fitted joint model we found the value association (95% credible interval in brackets) to be 0.182 [0.090, 0.274], and the velocity association to be -0.001 [-0.295, 0.254]. That is even though the schedule of biopsies depended upon observed PSA values it did not lead to a spurious velocity association. To check if we correctly specified the joint model, we performed several sensitivity analysis in our model (e.g., changing the position of the knots, etc.) to investigate the fit of the model and also the robustness of the results. In all of our attempts,

the same conclusions were reached, namely that the velocity of longitudinal outcome is more strongly associated with the hazard of GR compared to the value of longitudinal outcome.

Web Appendix D. Personalized Schedules for the Demonstration Patients from PRIAS.

In this section we demonstrate the application of personalized schedules on patients from PRIAS study. In Section 5.2 of the main manuscript we demonstrated personalized schedules for the first demonstration patient. Here we demonstrate them for the remaining two patients.

The evolution of PSA, repeat biopsy history and proposed times of biopsies for the second demonstration patient are shown in the top right and top left panels of Web Figure 4. It can be seen that the schedule of biopsy based on expected time of GR adjusts the times of biopsy according to the rise in hazard, which increases due to steep rise in $\log_2$ PSA velocity. More specifically, at year two the proposed biopsy time is 12.36 years whereas at year four it decreases to 3.94 years. On average, a biopsy scheduled using expected time of GR at year two should have a larger offset O_j^S compared to the same at year four. This is because the standard deviation of $g(T_j^*)$, given by $\mathrm{SD}_g(T_j^*) = \sqrt{\mathrm{var}_g(T_j^*)}$, is slightly lower at year four as shown in the bottom left panel of Web Figure 4. As for the schedules based on dynamic risk of GR, the threshold κ was automatically chosen using F_1 score, and was estimated to be between 1 and 0.9 at all time points. This value of κ corresponds to a time very close to the time of latest biopsy ($t = 0$). Hence the biopsies are scheduled much earlier than those based on expected time of GR.

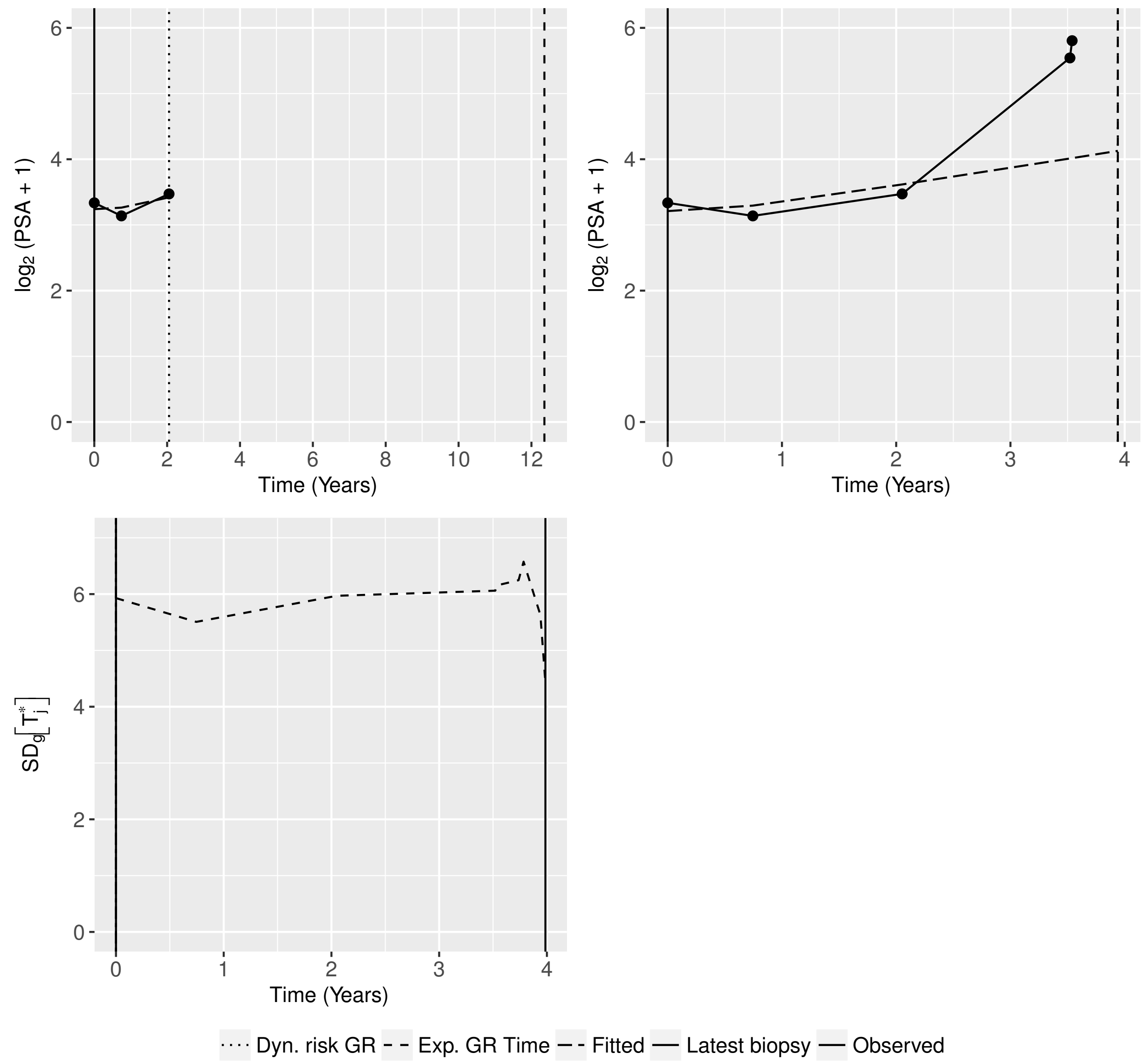

Web Figure 4. Top panel: Fitted versus observed $\log_2(\text{PSA}+1)$ profile, history of repeat biopsies and corresponding personalized schedules for the second demonstration patient. Bottom Panel: History of repeat biopsies and standard deviation $\text{SD}_g(T_j^*) = \sqrt{\text{var}_g(T_j^*)}$ of the posterior predictive distribution of time of GR over time for the second demonstration patient.

The third demonstration patient presents a case where information from PSA levels and repeat biopsies is not in concordance with each other. In Web Figure 5 we can see that the PSA for this patient increased by 100% between year two and year 3.2. If only information from PSA is considered, then we can see that proposed time of biopsy based on expected time of GR is preponed from 14.26 to 13.56 years during this period. However, if we also take into account the negative result from the repeat biopsy at year 2.5, then the proposed time of biopsy is postponed from 14.26 years to 15.15 years. Thus more weight is given to a recent negative biopsy result than PSA, which is in accordance with the clinical practice. The proposed time of biopsy based on dynamic risk of GR is also postponed from 2.27 to 3.55 years in light of the negative biopsy result.

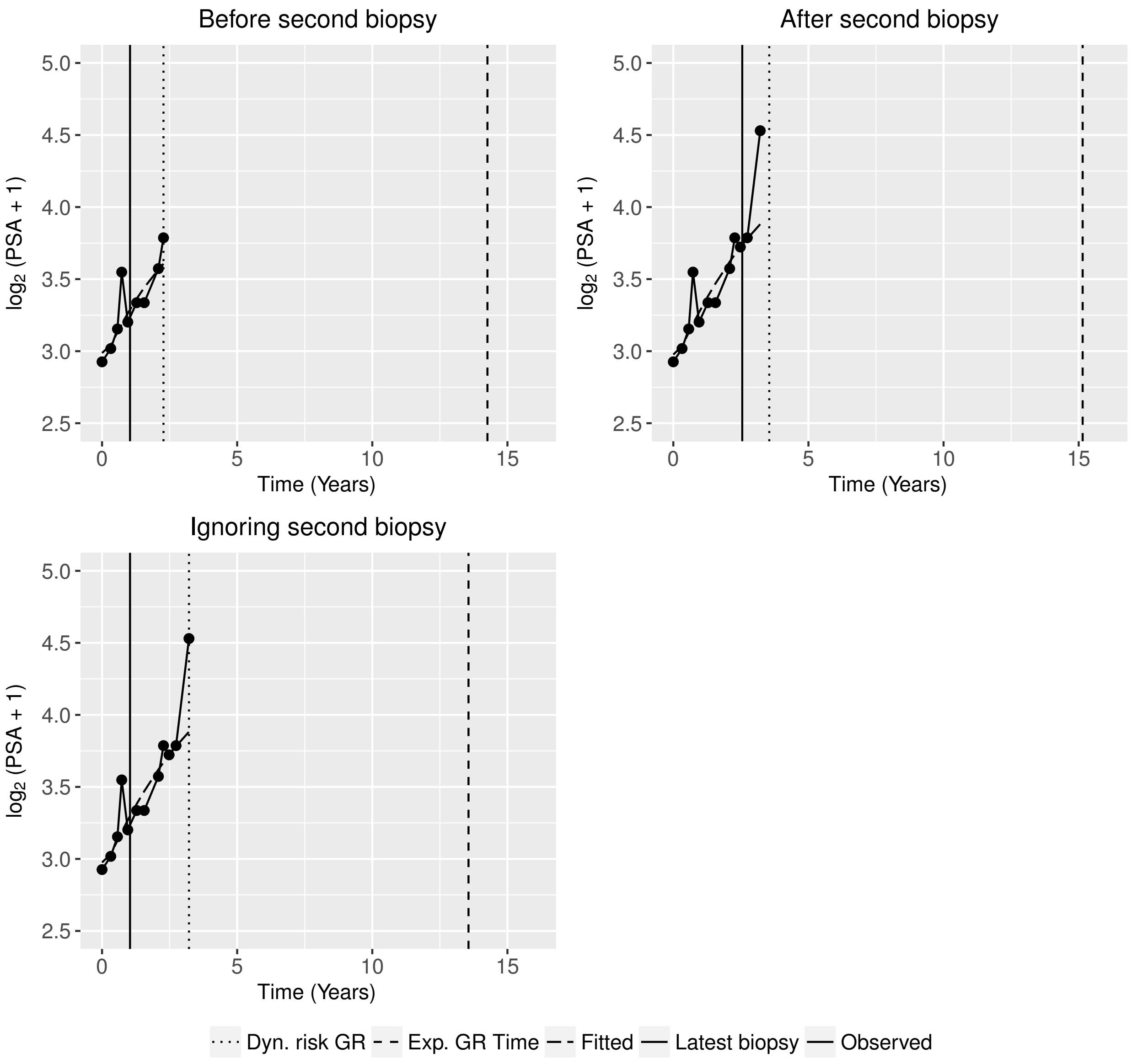

Web Figure 5. Fitted versus observed $\log_2(\text{PSA} + 1)$ profile, history of repeat biopsies and corresponding personalized schedules for the third demonstration patient.

Web Appendix E. Simulation Study

Web Appendix E.1 *Simulation Results for Dynamic Risk of GR Based Approach With a Fixed* $\kappa = 0.95$

In the main manuscript, for the personalized schedules based on dynamic risk of GR we chose κ on the basis of F_1 score. However while conducting the simulation study, we also tried a fixed κ of 0.95, which means that the next biopsy is scheduled at a time point where the dynamic risk of GR is 5%. The results for this approach are presented in Web Table 4. In the table, the abbreviation Dyn. risk GR (F_1 score) corresponds to personalized schedules based on dynamic risk of GR based approach, with κ chosen on the basis of F_1 score. The abbreviation Hybrid (F_1 score) corresponds to the hybrid approach between median time of GR and dynamic risk of GR (κ chosen on the basis of F_1 score).

Web Table 4

Estimated mean and standard deviation (SD), of the number of biopsies N_j^S conducted until Gleason reclassification (GR) is detected, and of the offset O_j^S (difference in time at which GR is detected and the true time of GR, in months), for the simulated (500 datasets) test patients, across different schedules and subgroups. Patients in subgroup G_1 have the fastest prostate cancer progression rate, whereas patients in subgroup G_3 have the slowest progression rate. Types of personalized schedules (full names in brackets): Exp. GR Time (expected time of GR), Med. GR Time (median time of GR), Dyn. risk GR (schedules based on dynamic risk of GR), Hybrid (a hybrid approach between median time of GR and dynamic risk of GR). Annual corresponds to a schedule of yearly biopsies and PRIAS corresponds to biopsies as per PRIAS protocol.

a) All hypothetical subgroups				
Schedule	$E(N_j^S)$	$E(O_j^S)$	$\text{SD}(N_j^S)$	$\text{SD}(O_j^S)$
Annual	5.24	6.01	2.53	3.46
PRIAS	4.90	7.71	2.36	6.31
Dyn. risk GR (F_1 score)	4.69	6.66	2.19	4.38
Hybrid (F_1 score)	3.75	9.70	1.71	7.25
Dyn. risk GR ($\kappa = 0.95$)	5.15	6.02	2.51	3.47
Med. GR time	2.06	13.88	1.41	11.80
Exp. GR time	1.92	15.08	1.19	12.11
b) Hypothetical subgroup G_1				
Schedule	$E(N_j^S)$	$E(O_j^S)$	$\text{SD}(N_j^S)$	$\text{SD}(O_j^S)$
Annual	4.32	6.02	3.13	3.44
PRIAS	4.07	7.44	2.88	6.11
Dyn. risk GR (F_1 score)	3.85	6.75	2.69	4.44
Hybrid (F_1 score)	3.25	10.25	2.16	8.07
Dyn. risk GR ($\kappa = 0.95$)	4.23	6.05	3.10	3.46
Med. GR time	1.84	20.66	1.76	14.62
Exp. GR time	1.72	21.65	1.47	14.75
c) Hypothetical subgroup G_2				
Schedule	$E(N_j^S)$	$E(O_j^S)$	$\text{SD}(N_j^S)$	$\text{SD}(O_j^S)$
Annual	5.18	5.98	2.13	3.47
PRIAS	4.85	7.70	2.00	6.29
Dyn. risk GR (F_1 score)	4.63	6.66	1.82	4.37
Hybrid (F_1 score)	3.68	10.32	1.37	7.45
Dyn. risk GR ($\kappa = 0.95$)	5.09	5.99	2.11	3.47
Med. GR time	1.89	12.33	1.16	9.44
Exp. GR time	1.77	13.54	0.98	9.83
d) Hypothetical subgroup G_3				
Schedule	$E(N_j^S)$	$E(O_j^S)$	$\text{SD}(N_j^S)$	$\text{SD}(O_j^S)$
Annual	6.20	6.02	1.76	3.46
PRIAS	5.76	7.98	1.71	6.51
Dyn. risk GR (F_1 score)	5.58	6.58	1.56	4.33
Hybrid (F_1 score)	4.32	8.55	1.26	5.91
Dyn. risk GR ($\kappa = 0.95$)	6.11	6.01	1.76	3.46
Med. GR time	2.45	8.70	1.15	6.32
Exp. GR time	2.27	10.09	0.99	7.47

Web Appendix E.2 *Variation in Estimated Mean and Standard Deviation, of Number of Biopsies and Offset Across the 500 Simulations*

In this section we present figures related to the simulation study results discussed in Section 6 of main manuscript. The figures we present next are population specific, i.e. subgroup level differentiation is not done.

- Variation in estimated mean across the 500 simulations, for number of biopsies and offset (difference in time at which Gleason reclassification or GR is detected and the true time of GR, in months) for different methods is shown in Web Figure 6 and Web Figure 7.
- Variation in estimated standard deviation across the 500 simulations, for number of biopsies and offset (difference in time at which Gleason reclassification or GR is detected and the true time of GR, in months) for different methods is shown in Web Figure 8 and Web Figure 9.

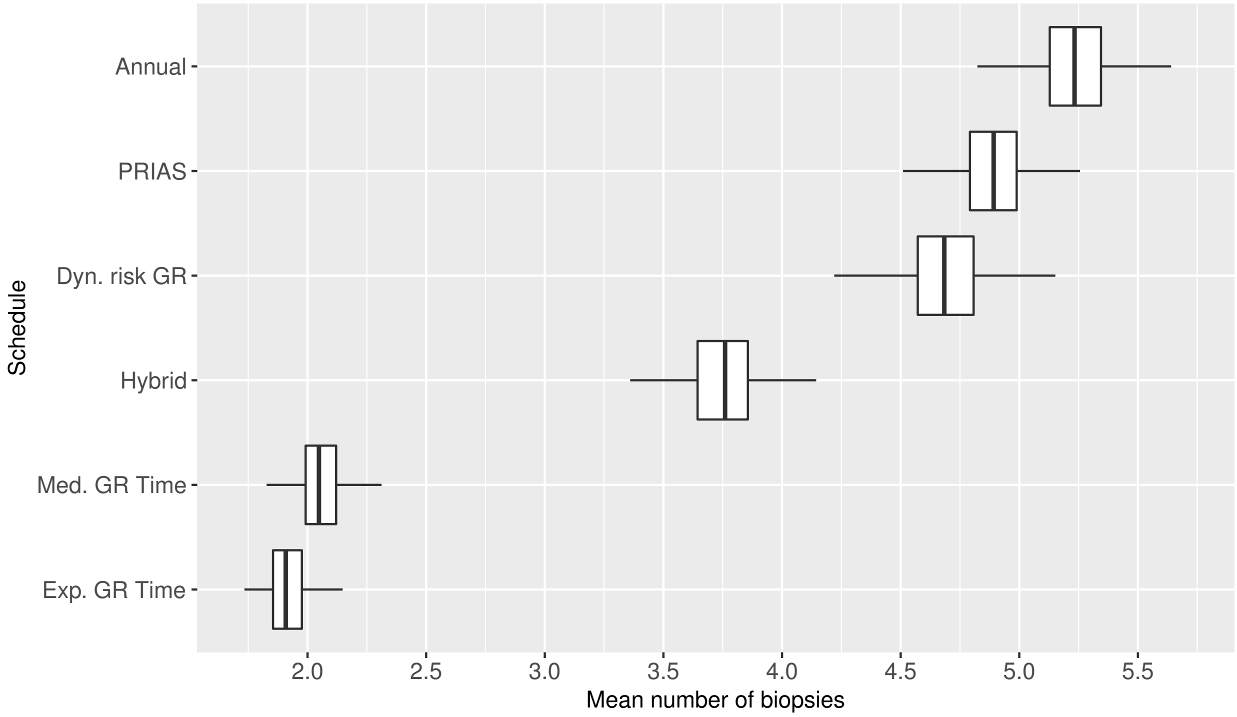

Web Figure 6. Boxplot showing variation in estimated mean number of biopsies conducted by various schedules until Gleason reclassification is detected, obtained from the simulation study with 500 simulated datasets.

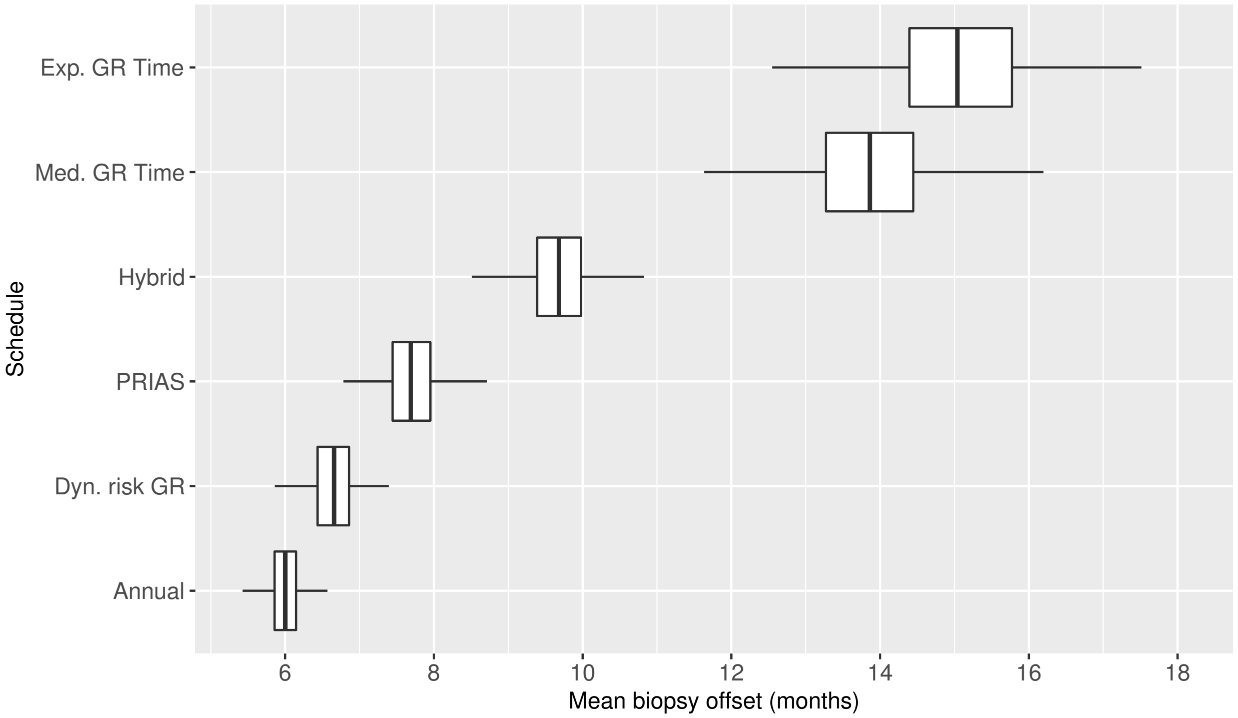

Web Figure 7. Boxplot showing variation in estimated mean of biopsy offset (difference in time at which Gleason reclassification or GR is detected and the true time of GR, in months) for various schedules, obtained from the simulation study with 500 simulated datasets.

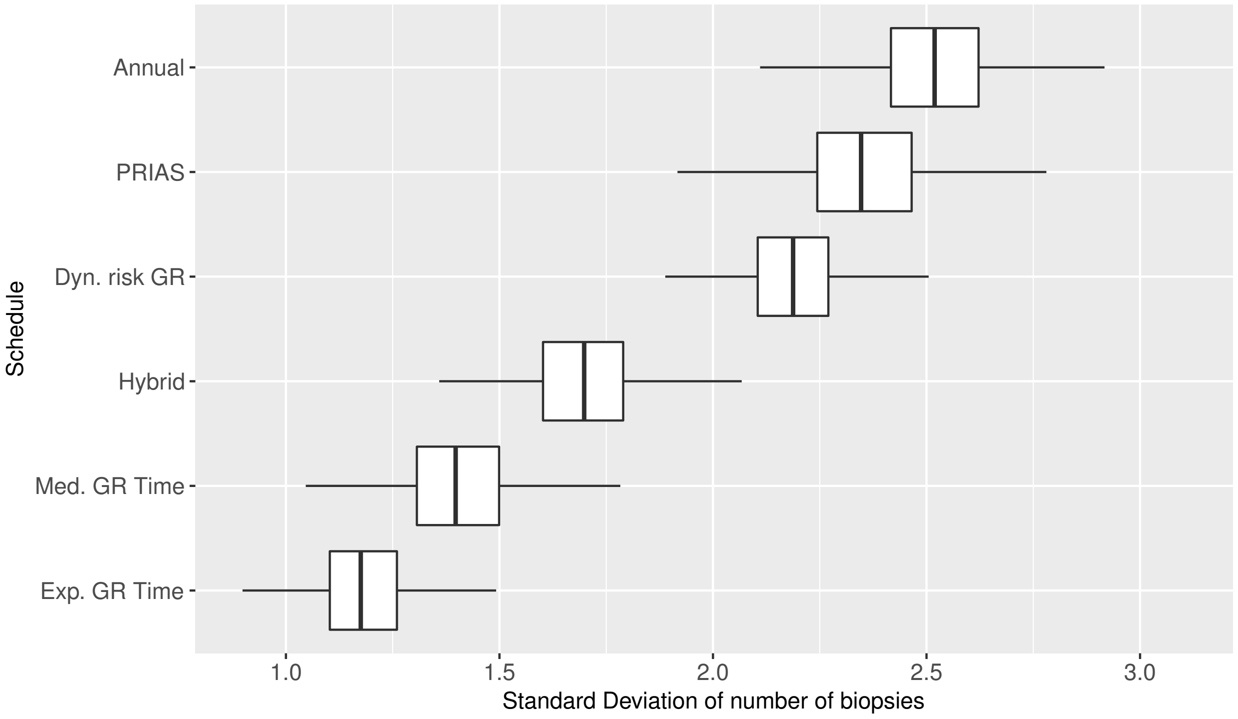

Web Figure 8. Boxplot showing variation in estimated standard deviation of number of biopsies conducted by various schedules until Gleason reclassification is detected, obtained from the simulation study with 500 simulated datasets.

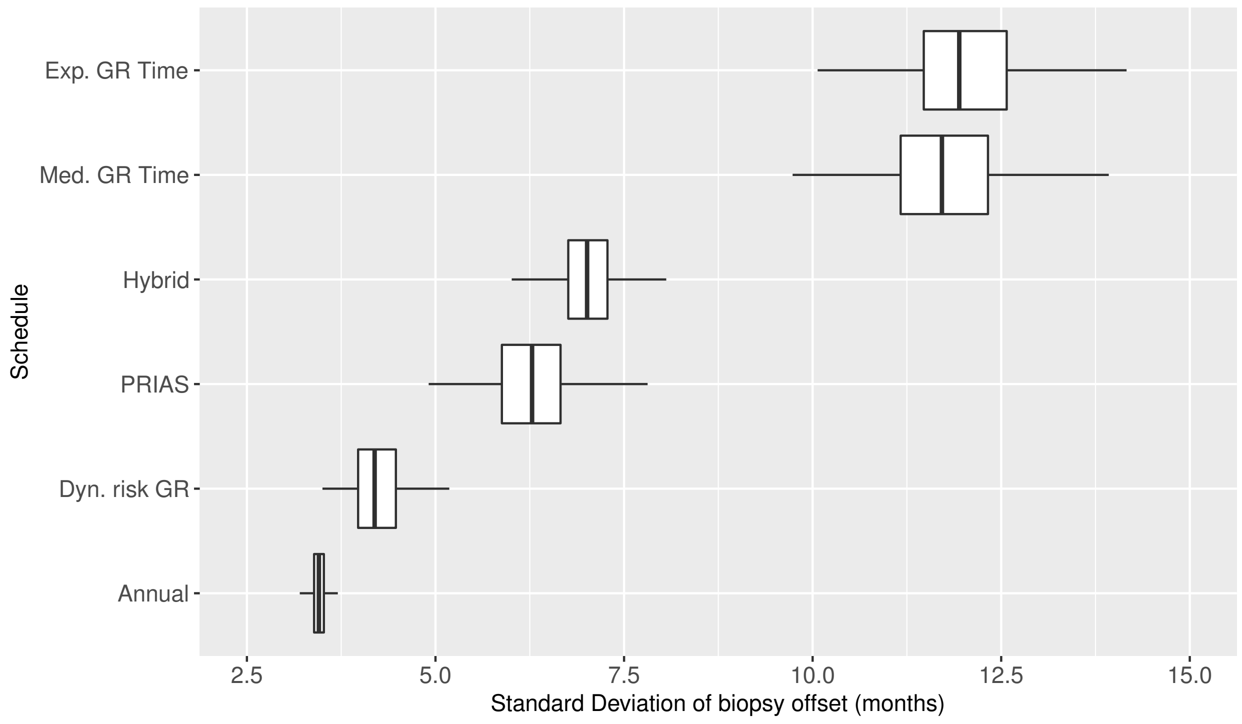

Web Figure 9. Boxplot showing variation in estimated standard deviation of biopsy offset (difference in time at which Gleason reclassification or GR is detected and the true time of GR, in months) for various schedules, obtained from the simulation study with 500 simulated datasets.

Web Appendix F. Source Code

The R code for fitting the joint model to the PRIAS dataset, and for the simulation study, along with sample dataset are available with this paper at the Biometrics website on Wiley Online Library.

References

Brown, E. R. (2009). Assessing the association between trends in a biomarker and risk of event with an application in pediatric HIV/AIDS. *The Annals of Applied Statistics* **3,** 1163–1182.

Eilers, P. H. and Marx, B. D. (1996). Flexible smoothing with B-splines and penalties. *Statistical Science* **11,** 89–121.

Gentleman, R. and Geyer, C. J. (1994). Maximum likelihood for interval censored data: Consistency and computation. *Biometrika* **81,** 618–623.

Lin, H., McCulloch, C. E., Turnbull, B. W., Slate, E. H., and Clark, L. C. (2000). A latent class mixed model for analysing biomarker trajectories with irregularly scheduled observations. *Statistics in Medicine* **19,** 1303–1318.

Pearson, J. D., Morrell, C. H., Landis, P. K., Carter, H. B., and Brant, L. J. (1994). Mixed-effects regression models for studying the natural history of prostate disease. *Statistics in Medicine* **13,** 587–601.

Rizopoulos, D. (2011). Dynamic predictions and prospective accuracy in joint models for longitudinal and time-to-event data. *Biometrics* **67,** 819–829.

Rizopoulos, D. (2012). *Joint Models for Longitudinal and Time-to-Event Data: With Applications in R.* CRC Press.

Rizopoulos, D., Hatfield, L. A., Carlin, B. P., and Takkenberg, J. J. (2014). Combining dynamic predictions from joint models for longitudinal and time-to-event data using Bayesian model averaging. *Journal of the American Statistical Association* **109,** 1385–1397.

Rizopoulos, D., Molenberghs, G., and Lesaffre, E. M. (2017). Dynamic predictions with time-dependent covariates in survival analysis using joint modeling and landmarking. *Biometrical Journal* doi:`10.1002/bimj.201600238`.

Taylor, J. M., Park, Y., Ankerst, D. P., Proust-Lima, C., Williams, S., Kestin, L., Bae, K., Pickles, T., and Sandler, H. (2013). Real-time individual predictions of prostate cancer recurrence using joint models. *Biometrics* **69,** 206–213.

Tsiatis, A. A. and Davidian, M. (2004). Joint modeling of longitudinal and time-to-event data: an overview. *Statistica Sinica* **14,** 809–834.

Received October 0000. *Revised February* 0000. *Accepted March* 0000.
